# Spatial constraints determine the spread of plasmid-encoded antibiotic resistance between bacterial colonies

**DOI:** 10.1128/msystems.00531-26

**Published:** 2026-08-28

**Authors:** Josep Ramoneda, Deepthi P. Vinod, Yinyin Ma, Chujin Ruan, Julian Schmidt, Michael Manhart, Daniel C. Angst, David R. Johnson

**Affiliations:** 1 Department of Environmental Microbiology, Swiss Federal Institute of Aquatic Science and Technology (Eawag), Dübendorf, Switzerland; 2 Department of Ecology and Complexity, Center for Advanced Studies of Blanes (CEAB), Spanish Research Council (CSIC), Blanes, Spain; 3 Department of Environmental Systems Science, Swiss Federal Institute of Technology (ETH) 28499, Zürich, Switzerland; 4 Center for Advanced Biotechnology and Medicine and Department of Biochemistry and Molecular Biology, Rutgers University Robert Wood Johnson Medical School 332317, Piscataway, New Jersey, USA; 5 Institute of Ecology and Evolution, University of Bern 27210, Bern, Switzerland; University of Minnesota Twin Cities, St. Paul, Minnesota, USA; University of Liverpool, Liverpool, United Kingdom

**Keywords:** plasmid dynamics, horizontal gene transfer, spatial self-organization, antibiotic resistance, microbial interactions, individual-based modelling, biofilms

## Abstract

Plasmid transfer among bacteria is an important driver of the spread of antibiotic resistance. Surface-associated bacterial biomass is a hotspot for plasmid transfer due to the dense spatial packing of cells, but this biomass is often sparse (composed of discrete bacterial colonies). Compared to plasmid dynamics within a single colony, the determinants of plasmid transfer between discrete colonies are less understood. Yet, colonies routinely physically collide with each other as they grow and expand across surfaces. Here, we experimentally demonstrate that collisions between colonies of *Stutzerimonas stutzeri* and *Escherichia coli* enable the spread of an antibiotic resistance-encoding plasmid, with the extent of transfer determined by the spatial distance between bacterial inocula. To better understand how spatial constraints influence the mechanisms underlying inter-colony plasmid spread, we applied an individual-based model simulating plasmid dynamics between colliding colonies. Our simulations quantitatively predict how the probabilities of plasmid transfer and loss affect plasmid spread as colonies grow and collide. These effects are modulated by the distances between colonies and the spatial positioning of plasmid-carrying cells along the collision boundary. Our study reveals that inter-colony plasmid transfer is determined by the interplay between plasmid transfer, plasmid loss, and spatial constraints, expanding our understanding of plasmid dynamics in the spread of antibiotic resistance genes.

**IMPORTANCE:**

The spread of antibiotic resistance between spatially discrete microbial colonies is poorly understood, despite its relevance to persistent colonization on a variety of surfaces (e.g., medical devices, dental plaque, wound infections, indoor plumbing, etc.). Here, we combined experiments and individual-based modeling to show that physical collisions between growing colonies enable the spread of plasmids carrying antibiotic resistance genes. The extent of transfer depends on the initial spatial distance between colonies, the probabilities of plasmid transfer and loss, and the local spatial intermixing of plasmid-carrying and -free cells along the collision boundary. These findings reveal how the interplay between plasmid biology and microbial spatial organization governs the spread of antibiotic resistance and provide a quantitative framework for predicting plasmid dynamics in spatially structured environments.

## INTRODUCTION

Surface-associated bacterial biomass, such as biofilms, cell aggregates, and colonies, is pervasive on our planet (1), drives major biogeochemical cycles (2, 3), and affects human health and disease (4–6). They are engineered for diverse biotechnological applications, such as water decontamination and biofuel production (7, 8), but also colonize animal and plant tissues (9), emerging particulate pollutants, such as microplastics (10), and medical devices (11). Within surface-associated biomass, the dense spatial packing and sessile state of bacterial cells can promote the contact-dependent horizontal transfer of plasmids and their associated genes (12). This has serious consequences for the emerging antibiotic resistance crisis, as antibiotic resistance-encoding plasmids can cause persistent resistant bacterial populations in human and environmental microbiomes, posing a threat to global health (13, 14). While important, our understanding of how the spatial features of (and spatial constraints acting on) surface-associated biomass direct the spread of antibiotic resistance-encoding plasmids remains in its infancy.

Plasmid dynamics within a single patch of surface-associated biomass, such as a colony, are determined by how easily plasmids transfer between and persist among the component populations. This is primarily controlled by the likelihood that cells acquire plasmids from each other when in close spatial proximity (i.e., the plasmid transfer probability), the likelihood that plasmids are lost during cell division (i.e., the plasmid loss probability), and the strength of selection on plasmid-encoded traits (15, 16). Thus, plasmid loss can be offset by high plasmid transfer probabilities, leading to the persistence of antibiotic resistance even in the absence of positive selection (17). Since plasmid transfer and loss probabilities determine the load and distribution of plasmids over time, we expect these mechanisms to be important determinants of plasmid spread within surface-associated biomass.

In addition to nutrient availability due to host growth or nutrient pulses (18), the spatial arrangement of cells within a patch of surface-associated biomass is another important determinant of plasmid transfer and spread (19–21). During surface-associated growth, the component populations typically spatially segregate from each other as a consequence of stochastic drift along the biomass periphery (22). This process has important effects on diversity (22, 23), stability (24), and functioning (25). Plasmids transfer more extensively when the component populations are highly spatially intermixed, which can be ascribed to the greater number of cell-cell contacts between plasmid-free and -carrying cells that are necessary for plasmid transfer (18, 26, 27). Frequent disturbance can also promote plasmid transfer by causing the spatial reorganization of cells and creating new cell-cell contacts between otherwise spatially segregated populations (21, 28).

While the processes governing plasmid transfer within a single patch of surface-associated biomass are now becoming clear, surfaces are generally not colonized by a single contiguous biomass patch. Rather, they are colonized by many spatially discrete collections of cells (referred to as colonies hereafter) that collectively form a sparse biofilm, where each colony lies adjacent to others and dynamically expands and contracts in size as a consequence of growth and death (29, 30). Despite the pervasiveness of such sparse biofilms, there is little information on the processes affecting plasmid transfer between the component colonies (18, 31, 32). In one study, Lagido et al. (33) established a mathematical model demonstrating that collisions between adjacent colonies enable conjugative plasmid transfer, with the probability of contact and subsequent transfer depending on the initial distance between colonies. Yet, the dearth of information on this topic contrasts the pervasiveness of colony collisions, which likely occur on medical implants (34, 35), dental plaque (36, 37), and wound infections (38), but also in environmental settings such as in soils (39), water plumbing (40), and wastewater treatment systems (41). In these systems, periodic disturbances (e.g., by antibiotic administration in clinical settings or through physical alteration in natural settings) can disrupt contiguous biofilms. This can then increase biomass sparseness and exacerbate the subsequent spread of plasmid-encoded antibiotic resistance during growth and recovery (42). This spread can happen through direct positive selection pressure during antibiotic treatment (43, 44), as well as indirectly via additional horizontal gene transfer (HGT) on natural surfaces (45). Understanding the mechanisms governing the transfer of antibiotic resistance-encoding plasmids during physical collisions of discrete colonies after such disturbances, in particular the role of plasmid transfer and loss probabilities in relation to spatial features, would help address the emerging antibiotic resistance crisis by improving our ability to predict plasmid dynamics and fate.

In this study, we investigated the determinants of the horizontal transfer of an antibiotic resistance-encoding plasmid during the growth and physical collision of discrete bacterial colonies. We designed an experimental system in which a plasmid donor colony of the bacterium *Stutzerimonas stutzeri* A1601 (formerly *Pseudomonas stutzeri*; see reference 46), which carries the self-transmissible conjugative plasmid pAR145, grows and eventually collides into an *Escherichia coli* potential recipient colony. pAR145 encodes chloramphenicol resistance and cyan fluorescent protein, thus enabling us to track and quantify its intra- and inter-colony transfer across space and time. We can also use the system to quantify the extent of inter-colony plasmid transfer as a function of the initial distance between the growing colonies. To elucidate the mechanistic basis of our observations and interrogate parameters not easily manipulated experimentally, we adapted an individual-based model to simulate the growth and physical collision of discrete colonies. Using this model, we established quantitative predictions about how intra- and inter-colony plasmid transfer and plasmid loss probabilities jointly affect the spread of a conjugative plasmid between discrete colonies located at different initial distances from each other. We additionally used the model to investigate the influence of the single-cell-level spatial organization of the colonies on plasmid spread, enabling us to generalize our findings beyond the specific conditions tested experimentally.

## RESULTS

### Experimental quantification of plasmid dynamics during bacterial colony growth and collision

**Fig 1:**
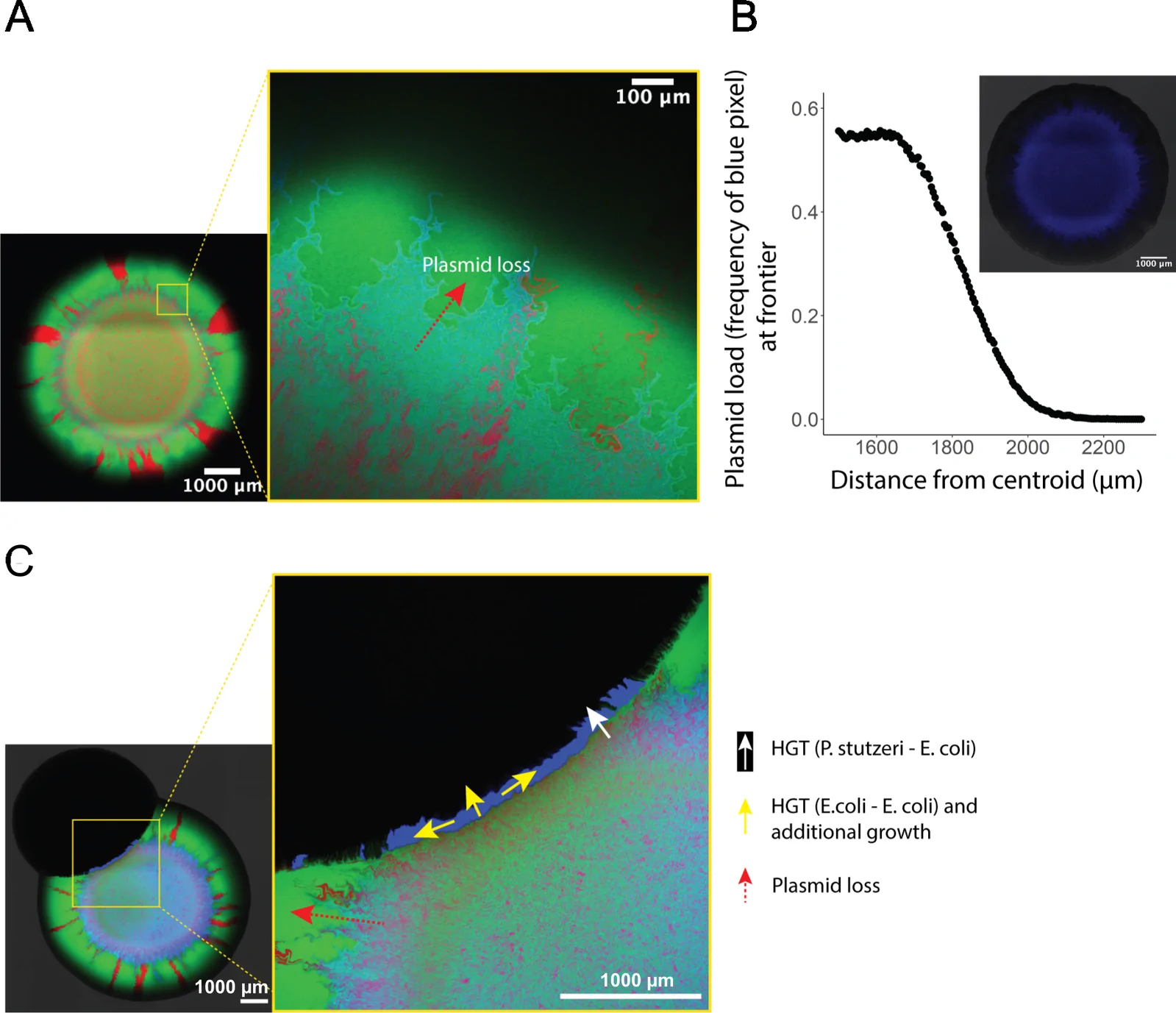
Plasmid dynamics in plasmid donor colonies and inter-colony transfer to potential recipient colonies upon collision. (**A**) Representative microscopy image of the *S. stutzeri* plasmid donor colony consisting of two isogenic strains. One strain expresses red fluorescent protein from the chromosome and cyan fluorescent protein from pAR145 and appears magenta. The other strain expresses green fluorescent protein from the chromosome and appears green. If pAR145 transfers into the strain expressing green fluorescent protein, then it appears cyan. (**B**) Quantification of pAR145 abundance (blue pixels) during growth of the plasmid donor colony shown in panel **A**. (**C**) Representative microscopy image of a collision between a *S. stutzeri* plasmid donor colony and a potential *E. coli* recipient colony. Note the formation of a blue patch located along the collision boundary, which corresponds to *E. coli* cells that acquired pAR145 via inter-colony transfer from pAR145-carrying *S. stutzeri* cells (white arrow). Yellow arrows indicate the spread of pAR145 into the recipient colony, which can happen via transfer among *E. coli* cells, as well as additional growth.

We developed an experimental system that allowed us to grow bacterial colonies across nutrient-amended surfaces, collide them into each other, and quantify plasmid dynamics during growth and collision. Briefly, we grew a plasmid donor colony containing two fluorescently labeled strains of *S. stutzeri*, where one carries pAR145 while the other does not, until it physically collided with a plasmid-free *E. coli* potential recipient colony. We first quantified pAR145 dynamics within the plasmid donor colony prior to its collision with the potential recipient colony, as we expected the processes of intra-colony pAR145 transfer and loss during the growth of the plasmid donor colony to affect the total load and thus transfer of pAR145 into the potential recipient colony. We found that pAR145 was nearly purged from the periphery of the plasmid donor colony after growing approximately 300 µm in the radial direction (corresponding to the radial interval between 1,700 and 2,000 µm) (Fig. 1A and B), which is the accumulated consequence of pAR145 loss and the faster growth of pAR145-free cells in the absence of chloramphenicol. Note that the intra-colony transfer of pAR145 readily occurred between plasmid-carrying and -free cells of *S. stutzeri* within the plasmid donor colony (see cyan areas in Fig. 1A). In our system, this radial interval between 1,700 and 2,000 µm determines the space/time where the intra-colony transfer of pAR145 to *E. coli* cells within the potential recipient colony is likely to occur upon collision with the plasmid donor colony, as it determines the space/time when the plasmid is present along the periphery of the plasmid donor colony.

### Inter-colony transfer of pAR145 between colliding colonies depends on the initial distance between inocula

We next experimentally tested whether physical collisions between the *S. stutzeri* plasmid donor and the *E. coli* potential recipient colonies can promote the inter-colony transfer of pAR145. To accomplish this, we placed a 1 µL drop of the *S. stutzeri* inoculum at ca. 3.0 mm from the *E. coli* inoculum. After 96 h of incubation, the plasmid donor and potential recipient colonies grew and physically collided into each other. We found that a new genotype formed along the collision boundary (Fig. 1C), which consisted of initially non-fluorescent *E. coli* potential recipient cells that obtained pAR145 from *S. stutzeri* plasmid donors upon colony collision. A closer evaluation of the collision boundary revealed that the newly formed *E. coli* transconjugants contiguously extended across an approximately 2 mm long boundary and uniformly protruded ca. 80 µm into the *E. coli* potential recipient colony (Fig. 1C).

**Fig 2:**
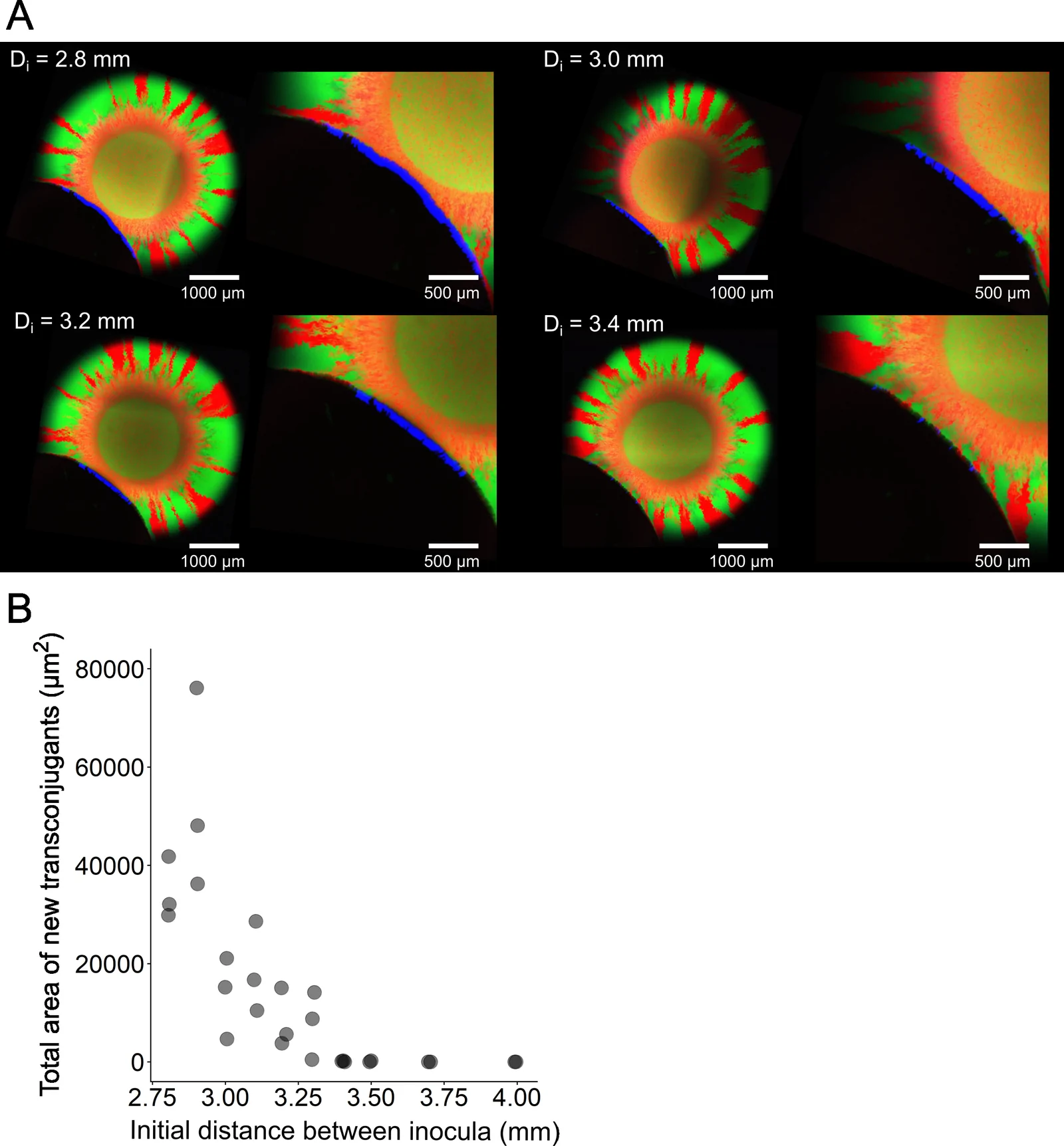
Initial distance between bacterial inocula determines the extent of inter-colony pAR145 transfer upon colony collision. (**A**) Representative microscopy images of experimental collisions between *S. stutzeri* plasmid donor and *E. coli* potential recipient colonies. Numbers indicate the distances between the centroids of the inocula. Inter-colony pAR145 transfer occurred along the collision boundaries and generated blue patches consisting of *E. coli* transconjugants carrying pAR145. Note that due to overexposure of the green and red channels, mixed populations of *S. stutzeri* do not display visible cyan signals even when carrying pAR145. *E. coli* does not express green or red fluorescent protein and thus appears blue when carrying pAR145. (**B**) Quantification of the absolute area of *E. coli* transconjugants as a function of the distance between the inocula. Each data point is for an independent biological replicate (*n* = 3) at the specified initial distance *D*_*i*_ (note that some data points are overlapping and thus appear as one).

After verifying the inter-colony transfer of pAR145 between *S. stutzeri* plasmid donor and *E. coli* potential recipient colonies upon their physical collision, we next investigated the effect of the initial distance between inocula on this process. Based on the simulations reported by Lagido et al. (33), we hypothesized that the initial distance between the plasmid donor and potential recipient inocula would determine the extent of inter-colony pAR145 transfer, where larger distances increase the time for pAR145 loss to occur from the plasmid donor colony prior to its collision with the potential recipient colony, and thus reduce the inter-colony transfer of pAR145. This is mainly caused by plasmid maintenance costs and the selective pressures acting on plasmid-encoded traits (47, 48). To test this hypothesis, we used robotics to deposit the *S. stutzeri* plasmid donor and *E. coli* potential recipient inocula at precisely defined distances from each other and allowed them to develop into colonies that grew and eventually collided into each other. We found that collisions between colonies initially inoculated at closer distances led to larger amounts of new *E. coli* transconjugants (and thus more inter-colony transfer of pAR145) upon collision than did colonies initially inoculated at longer distances (Fig. 2A). The spatial range in which new *E. coli* transconjugants formed was for initial distances between 2.8–3.3 mm, and the amount of new *E. coli* transconjugants decreased monotonically within this range (Spearman rank correlation test; rho = −0.793, *P* < 0.001, *N* = 30) (Fig. 2B).

### Individual-based modeling framework recapitulates and generalizes experimental observations of inter-colony plasmid transfer

**Fig 3:**
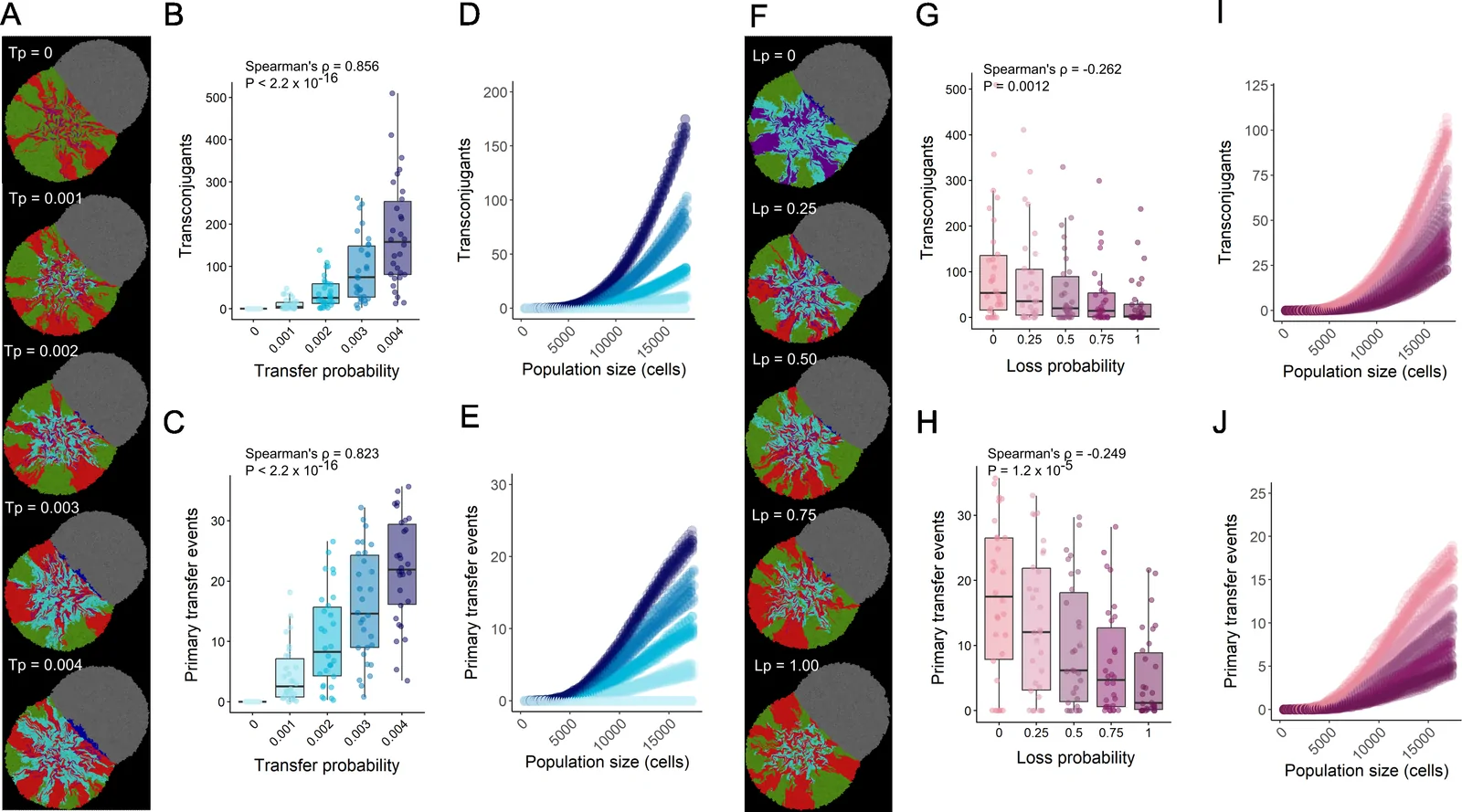
Effect of plasmid transfer and loss probabilities on plasmid spread between colliding colonies. (**A and F**) Representative individual-based simulations where the initial inoculum for the plasmid donor colony consisted of a 1:1 mixture of plasmid-carrying (magenta) and -free (green) cells and the inoculum for the potential recipient colony consisted of a single population of plasmid-free cells (gray) that can receive the plasmid upon colony collision (blue). Plasmid-free cells in the donor colony become cyan when they receive the plasmid. Tp and Lp values at the top left of the simulation images are the plasmid transfer and loss probabilities for the simulations in panels **A** and **F**, respectively. (**B**) Total number of transconjugants (blue) at the end of the simulations as a function of the plasmid transfer probability. (**C**) Total number of primary plasmid transfer events between the plasmid donor and potential recipient colonies at the end of the simulations as a function of the plasmid transfer probability. (**D**) Total number of transconjugants (blue) as a function of the total population size, colored by the plasmid transfer probability as defined in panel **B**. (**E**) Total number of primary plasmid transfer events between the plasmid donor and potential recipient colonies as a function of the total population size, colored by the plasmid transfer probability as defined in panel **B**. (**G**) Total number of transconjugants (blue) at the end of the simulations as a function of the plasmid loss probability. (**H**) Total number of primary plasmid transfer events between the plasmid donor and potential recipient colonies at the end of the simulations as a function of the plasmid loss probability. (**I**) Total number of transconjugants (blue) as a function of the total population size, colored by the plasmid loss probability as defined in panel **G**. (**J**) Total number of primary plasmid transfer events between the plasmid donor and potential recipient colonies as a function of the total population size, colored by the plasmid loss probability as defined in panel **G**. For panel **A**, there is a probability of 0.5 at each cell division that plasmid-carrying cells will lose the plasmid. For panel **F**, there is a probability of 0.003 that plasmid-carrying cells will transfer the plasmid to adjacent plasmid-free cells upon contact. Statistics in panels **B**, **C**, **G**, and **H** are for Spearman rank-correlation tests with the plasmid transfer probability (**B and C**) or plasmid loss probability (**G and H**) as the sole explanatory variable (*n* = 10).

Given the observed importance of plasmid loss and spatial factors in inter-colony plasmid transfer, we sought to quantitatively evaluate a broader range of plasmid and spatial parameters to test the generality of our findings. We experimentally calibrated the individual-based biophysical modeling framework of CellModeller (49) to test the influence of plasmid transfer and loss probabilities and spatial factors on intra- and inter-colony plasmid spread during the growth and collision of discrete bacterial colonies in a full factorial manner (transfer probability × loss probability × distance). Specifically, we simulated two colonies composed of distinct cell types: a plasmid donor colony composed of a 1:1 mixture of plasmid-carrying and -free cells, and a potential recipient colony composed solely of plasmid-free cells. For our simulations, we set the plasmid-carrying cells of the plasmid donor colony to have a 17% lower growth rate than the plasmid-free cells (referred to as the plasmid cost), which is based on our experimental measurements of the reduction in the growth rate caused by carrying the pAR145 plasmid (Table S1; Fig. S1 and S2). We applied this plasmid cost to all transconjugants emerging in the plasmid donor colony. We did not apply a plasmid cost to transconjugants emerging in the potential recipient colony, which is again based on our experimental measurements (Fig. S3). Using this modeling approach, we could successfully quantify plasmid dynamics in the plasmid donor colony and capture the formation of transconjugants within the potential recipient colony upon colony collision (Fig. 3A and F).

### Contributions of plasmid transfer and loss probabilities to inter-colony plasmid transfer

We used the total number of transconjugants in the potential recipient colony and the total number of plasmid transfer events between cells within the plasmid donor and potential recipient colonies (referred to hereafter as “primary transfer events”) as descriptors of plasmid spread. We also quantified plasmid transfer between transconjugants and plasmid-free cells in the potential recipient colony (referred to hereafter as “secondary plasmid transfer events”). We quantified these descriptors at the same final population size across all simulations. Both the plasmid transfer and loss probabilities significantly affected the total number of transconjugants and primary transfer events (Fig. 3A and F; Table S2). The plasmid transfer probability was the strongest overall driver, exerting a significant direct positive effect on the number of transconjugants (negative binomial generalized linear model [GLM]; *b* = 614.050, SE = 183.63, *P* = 0.001; Fig. 3B; Fig. S4) and a significant positive effect on primary transfer events mediated through its interaction with distance (*b* = 4.839, SE = 1.134, *P* < 0.001) (Fig. 3C; Fig. S5). The effect of the plasmid loss probability on the number of transconjugants was conditional on its interaction with distance (*b* = −0.036, SE = 0.009, *P* < 0.001; Fig. 3G; Fig. S6), with no significant independent main effect (*b* = 0.266, SE = 0.942, *P* = 0.777). Despite the absence of a significant main effect, the plasmid loss probability negatively influenced the overall transconjugant number, as reflected by a significant average marginal effect (AME = −100 transconjugants per unit increase, SE = 41.2, *P* = 0.015) and a significant descending monotonic trend in the data (Spearman’s ρ = −0.262, *P* = 0.001; Fig. 3G). By contrast, the plasmid loss probability exerted a significant direct positive main effect on the number of primary transfer events (*b* = 1.913, SE = 0.720, *P* = 0.008), additionally modulated by its interaction with distance (*b* = −0.050, SE = 0.007, *P* < 0.001) (Fig. 3H; Fig. S7; Table S2).

We verified that these effects remain valid when analyzing the data at a single common simulation time step of 640 rather than a single common population size (Fig. S8; Table S3). The number of secondary plasmid transfer events within the potential recipient colony exhibited the same pattern as the number of transconjugants; there was a significant positive effect of the plasmid transfer probability (*b* = 656.720, SE = 208.300, *P* = 0.002; Fig. S9A), a significant negative effect of the plasmid loss probability through its interaction with distance (*b* = −0.030, SE = 0.011, *P* = 0.006; Fig. S9C), and no significant independent main effect of the plasmid loss probability (*b* = −0.247, SE = 1.100, *P* = 0.823) despite showing a significant descending monotonic trend (Fig. S9C; Table S4). We also found that conditions of low plasmid transfer and high plasmid loss probabilities were conducive for the appearance of rare plasmid spread events in collisions occurring at large initial distances (Fig. S10A; Fig. S11A). By contrast, conditions of high plasmid transfer and low plasmid loss probabilities in collisions occurring at short initial distances led to highly predictable plasmid spread but with high absolute variance among replicates (Fig. S10A and S11A).

We next tracked the formation of new transconjugants during colony growth over time. The shape of the increasing trend in the total number of transconjugants throughout the simulations was strongly dependent on the plasmid transfer probability (Fig. 3D and I). When we evaluated the number of primary plasmid transfer events along the collision boundary, we found that it increased linearly from the beginning of colony growth but had a decelerating trend beginning at a colony size of ca. 12,000 cells independent of the plasmid transfer and loss probabilities (Fig. 3E and J). Over time, the accumulation of secondary plasmid transfer events had qualitatively similar increasing trends as the accumulation of new transconjugants (Fig. S9B and D).

### Effect of initial distance between colliding colonies on plasmid spread

**Fig 4:**
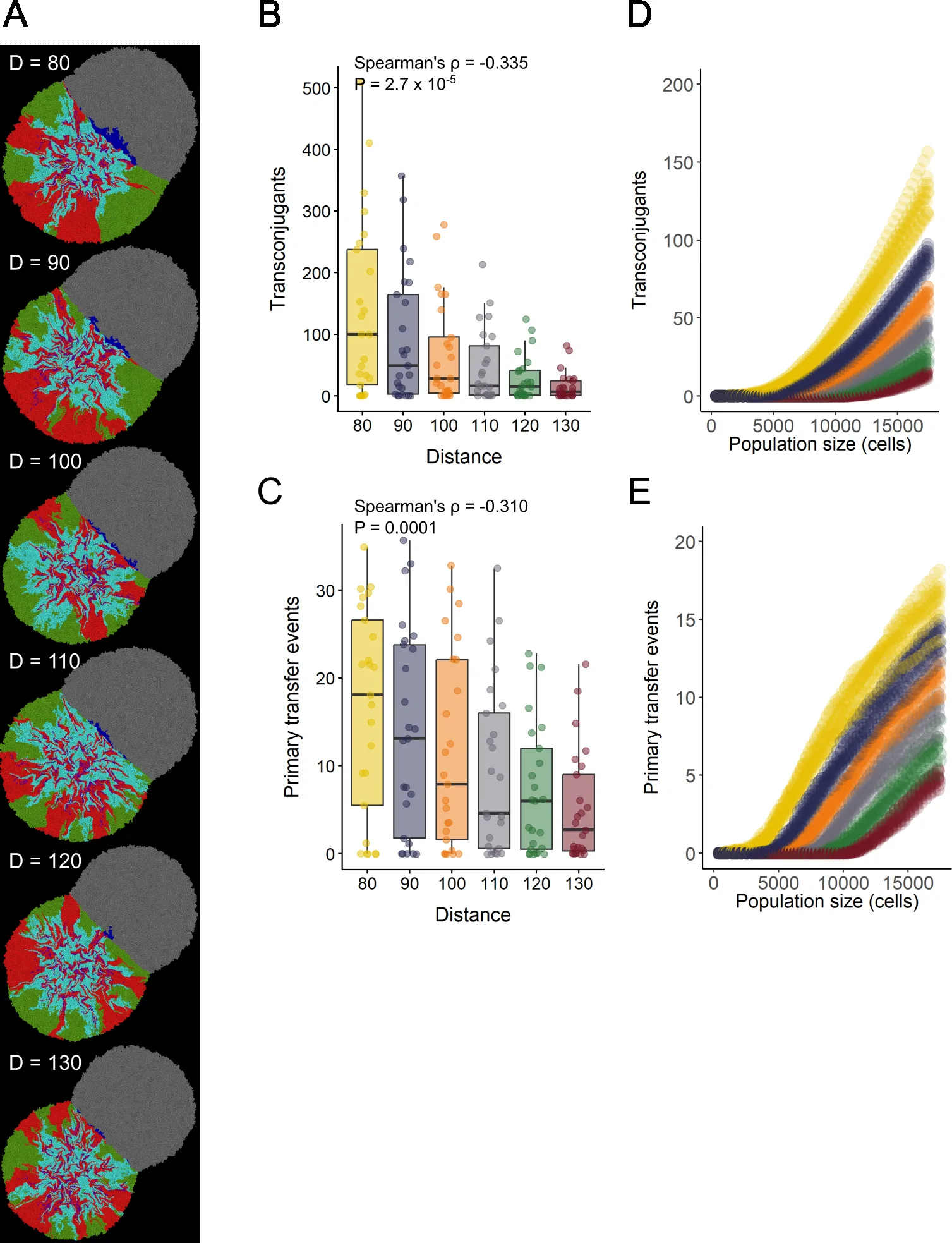
Effect of the initial distance between inocula on plasmid spread upon colony collision. (**A**) Representative individual-based simulations where the initial inoculum for the plasmid donor colony consisted of a 1:1 mixture of plasmid-carrying (magenta) and -free (green) cells and the inoculum for the potential recipient colony consisted of a single population of plasmid-free cells (gray) that can receive the plasmid upon colony collision (blue). *D*_*i*_ values at the top left of the simulation images are the initial distances between the centroids of the inocula. (**B**) Total number of transconjugants (blue in panel **A**) at the end of the simulations as a function of the initial distance (*D*_*i*_) between inocula. (**C**) Total number of primary plasmid transfer events between the plasmid donor and potential recipient colonies at the end of the simulations as a function of the initial distance (*D*_*i*_) between inocula. (**D**) Total number of transconjugants (blue in panel **A**) as a function of the total population size. (**E**) Total number of primary plasmid transfer events between the plasmid donor and potential recipient colonies as a function of the total population size. Colors are from panels **B** and **C**. For panel **A**, there is a probability of 0.5 at each cell division that a plasmid-carrying cell will lose its plasmid and a probability of 0.003 that a plasmid-carrying cell will transfer the plasmid to an adjacent plasmid-free cell upon contact. Statistics in panels **B** and **C** are for Spearman rank-correlation tests with initial distance as the sole explanatory variable (*n* = 10).

Using our modeling framework, we quantified the effect of the initial distance between the plasmid donor and potential recipient colonies on plasmid spread. We found that the initial distance had a strong negative effect on plasmid spread, where shorter distances increased both the total number of transconjugants and the number of primary transfer events (Fig. 4A). Distance had a stronger negative direct effect on the number of transconjugants (*b* = −0.038, SE = 0.005, *P* < 0.001; Fig. 4B) than on primary transfer events (*b* = −0.028, SE = 0.003, *P* < 0.001; Fig. 4C), with an additional effect on both outcomes mediated through its interaction with the plasmid loss probability (transconjugants: *b* = −0.036, SE = 0.009, *P* < 0.001; primary transfer events: *b* = −0.050, SE = 0.007, *P* < 0.001) (Fig. S6 and S7; Table S2). We verified that these effects remain valid when analyzing the data at a single common simulation time step of 640 rather than at a single common population size (Fig. S12; Table S3). The effect of initial distance on secondary plasmid transfer events was comparable, with a significant negative direct effect (*b* = −0.047, SE = 0.006, *P* < 0.001; Fig. S11A) and a significant interaction with the plasmid loss probability (*b* = −0.030, SE = 0.011, *z* = −2.743, *P* = 0.006).

The initial distance between inocula had a small effect on the shape of the curve describing the accumulation of new transconjugants or primary plasmid transfer events over time. The total number of new transconjugants increased in a continuous and non-decelerating manner throughout the simulations regardless of the initial distance between inocula (Fig. 4D), while the total number of primary plasmid transfer events had a slightly decelerating trend beginning at a colony size of ca. 12,000 cells (Fig. 4E). The temporal trend of accumulation of secondary plasmid transfer events in the potential recipient colony was again nearly exponential as observed for the accumulation of transconjugants (Fig. S13B).

### Initial distance determines the relative importance of plasmid transfer and loss on plasmid spread

**Fig 5:**
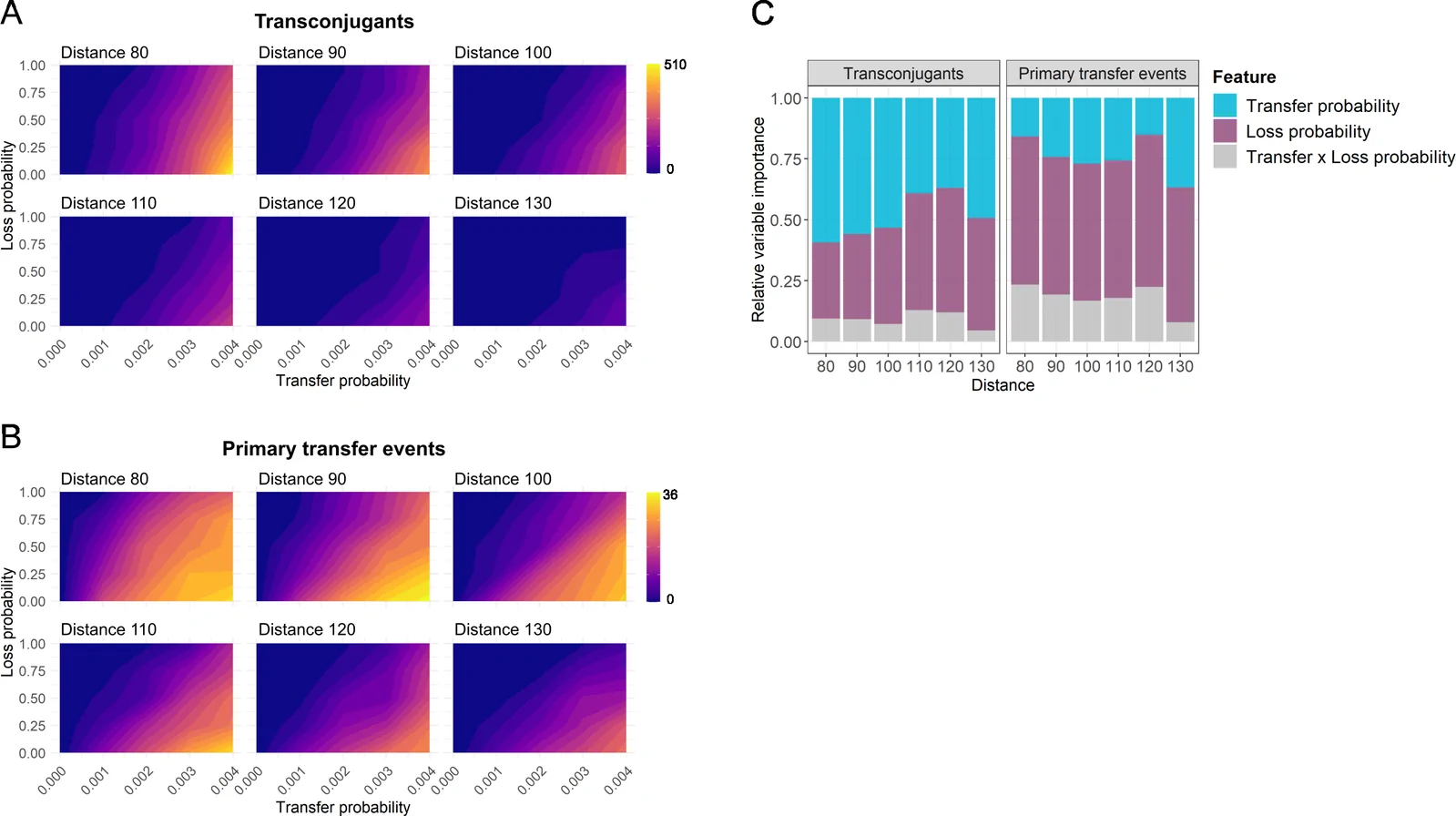
Interactive effects of the plasmid transfer and loss probabilities with the initial distance between inocula on plasmid spread upon colony collision. Contour plots showing the interaction between the plasmid transfer probability, plasmid loss probability, and initial distance between inocula on (**A**) the total number of transconjugants and (**B**) the total number of primary plasmid transfer events after collision of a plasmid donor and a potential recipient colony using an individual-based model. (**C**) Relative importance of the plasmid transfer and loss probabilities on plasmid spread as a function of the initial distance between inocula (*n* = 10).

Given the strong interactive effects detected of the initial distance between colonies with the plasmid transfer and loss probabilities in determining plasmid spread, we sought to quantitatively describe these effects. Longer initial distances require longer times for colonies to collide, which provides more time to accumulate plasmid transfer and loss events within the plasmid donor colony. The plasmid transfer probability was the main determinant of the total number of transconjugants (Fig. 5A), but this dominance was modulated by the initial distance between inocula. Specifically, the transfer probability decreased in importance with distance, explaining from 59.3% of the variation at the lowest initial distance of 80 to 36.9% at an initial distance of 120 (Fig. 5C). The relative importance of the interaction between plasmid transfer and loss probabilities was not affected by the initial distance. The relative importance of the plasmid loss probability went from 31.3% at an initial distance of 80 to 51.2% at an initial distance of 120 (Fig. 5C).

By contrast, the interactive effects of these factors with distance had the opposite trends in determining the total number of primary transfer events. The plasmid loss probability was the main determinant of the total number of primary transfer events (Fig. 5B), and this effect was modulated by the initial distance between inocula. Specifically, the plasmid transfer probability increased in importance with distance, explaining from 15.8% of the variation at the lowest initial distance of 80 to 36.7% at an initial distance of 130 (Fig. 5C). The relative importance of the interaction between the plasmid transfer and loss probabilities was again not affected by the initial distance. The relative importance of the plasmid loss probability in explaining the total number of primary transfer events went from 60.8% at an initial distance of 80 to 55.4% at an initial distance of 120 (Fig. 5C). For the total number of secondary plasmid transfer events within the recipient colony, the relative importance of the plasmid transfer probability had a moderate increase and that of the plasmid loss probability consequently decreased with the initial distance, with the relative importance of the plasmid transfer probability always being >50% (Fig. S14).

### Local spatial intermixing determines plasmid spread upon colony collision

**Fig 6:**
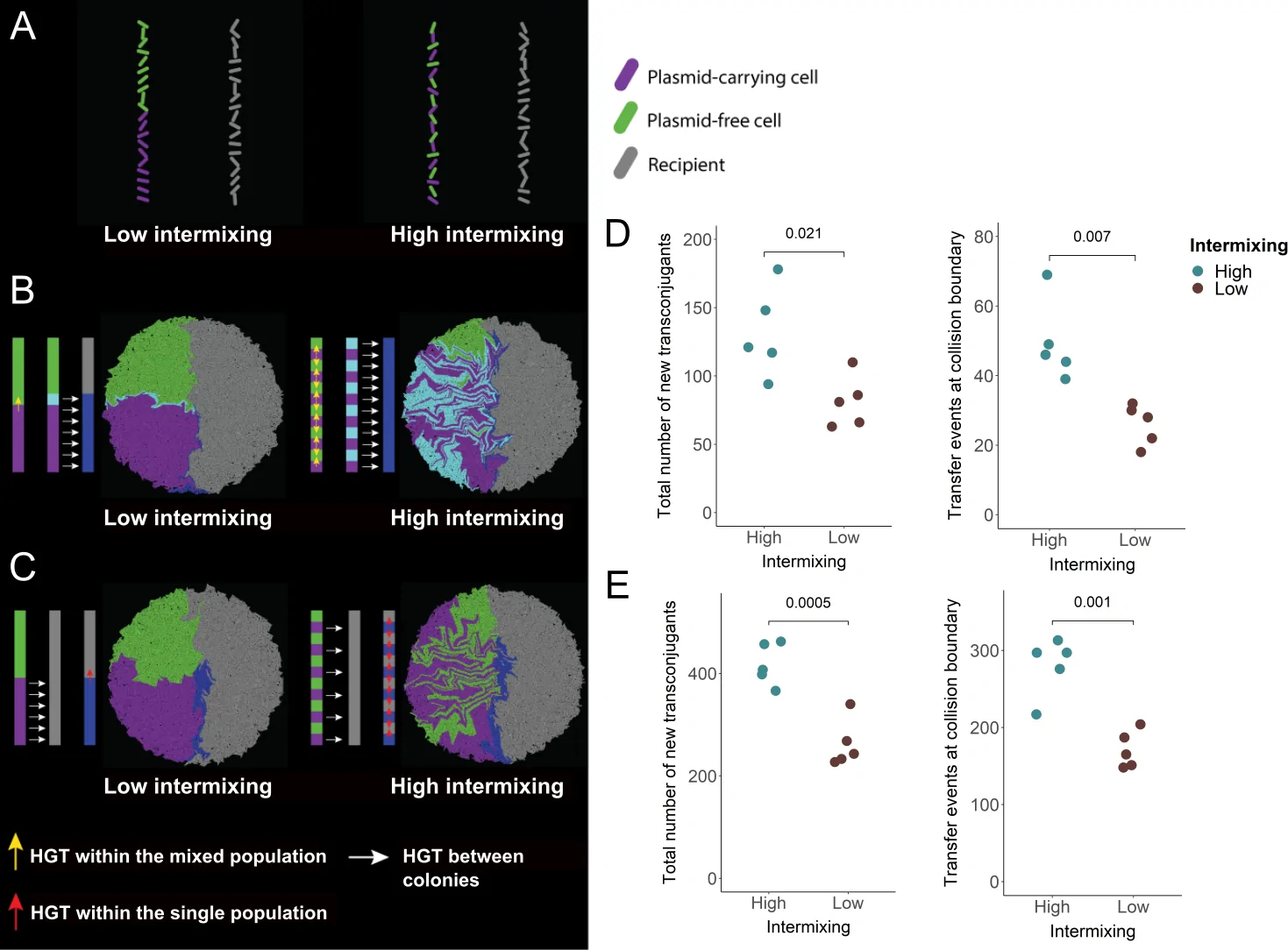
Effect of spatial intermixing of plasmid-carrying and -free cells along the collision boundary on plasmid spread. (**A**) Initial positions of cells along two parallel contiguous cell rows that eventually collide. Plasmid-carrying (magenta) and -free (green) cells within the plasmid donor cell row are distributed as either two discrete groups (low intermixing) or in alternating sequence (high intermixing). Potential recipient cells are gray and transconjugants in the potential recipient cell row are blue. (**B**) Simulation images where plasmid transfer was allowed within the donor cell row but not among cells in the recipient cell row. (**C**) Simulation images where plasmid transfer was allowed within the recipient cell row but not among cells in the donor cell row. Schematic illustrations of the expected effects are on the left, and the simulation outputs are on the right in panels **B** and **C**. As cell rows expand in all directions, these initially collide, and plasmids transfer from the donor to recipient cell row. Growth then proceeds in a radial manner due to the dense packing of cells at the center, thus forming a circular colony with two distinct hemispheres. (**D**) Quantification of the total number of transconjugants formed in the potential recipient cell row for the simulations in panel **B**. (**E**) Quantification of the total number of primary plasmid transfer events from the donor to the potential recipient cell row for the simulations in panel **C**. Each data point is for an independent simulation (*n* = 5). *P* values on the top of panels **D** and **E** are for two-sample two-sided Welch *t*-tests.

We finally hypothesized that the spatial organization of individual cells within the growing colonies would also be an important determinant of plasmid spread upon colony collision. More intermixed plasmid-carrying and -free populations can enable more efficient plasmid transfer than conditions where populations are spatially segregated (19, 20), as the number of contacts between plasmid-carrying and -free cells is higher when they are intermixed. In our system, as the initial distance between the inocula increases, we expect plasmid-carrying and -free cells within the plasmid donor colony to become increasingly spatially segregated due to longer growth times (as reported in references 19 and 20). This, in turn, should reduce the extent of plasmid transfer both within the potential recipient colony during its growth (intra-colony secondary plasmid transfer) and between colonies after their collision (inter-colony primary plasmid transfer). We used our individual-based modeling framework to simulate the collision boundary as two cell rows, where the plasmid donor cell row consisted of plasmid-carrying (magenta) and -free (green) cells and the recipient cell row consisted of a different population of plasmid-free (gray) cells (Fig. 6A). We varied the initial spatial intermixing of cells within the plasmid donor cell row by either placing the plasmid-carrying and -free cells in two discrete groups (low spatial intermixing) or by sequentially alternating a cell of one type next to a cell of the other (high spatial intermixing) (Fig. 6A). After the cell rows collide, a single circular colony forms where the left and right hemispheres contain information regarding plasmid spread from the plasmid donor to the potential recipient cell row. This simulation design allowed us to precisely control the spatial intermixing and organization of individual cells immediately before the point of collision and to investigate mechanisms occurring along the collision boundary.

We first tested the expectation that the extent of spatial intermixing will determine the total number of possible plasmid transfer events from the plasmid donor cell row into the potential recipient cell row. Higher spatial intermixing will increase the number of plasmid-carrying donor cells, as well as reduce the total number of redundant plasmid transfer events (transfer occurring between donor cells and recipient cells that already have the plasmid). To test this, we simulated two scenarios to isolate the effects of spatial intermixing within the donor cell row from those of spatial intermixing in the recipient cell row. Thus, we first allowed plasmid transfer to occur within the plasmid donor cell row and from the plasmid donor to the potential recipient cell row, but not within the potential recipient cell row (Fig. 6B). For collisions where plasmid-carrying cells (magenta) are highly intermixed with plasmid-free cells (green) in the plasmid donor cell row, we found that most of the plasmid-free cells (green) become plasmid-carrying cells (cyan) due to intra-row plasmid transfer, increasing the plasmid load along the collision boundary (Fig. 6B). As a consequence, both the total number of new transconjugants (blue) and the number of inter-row plasmid transfer events were higher for high spatial intermixing than for low spatial intermixing (two-sample two-sided Welch test; *P*_Transconjugants_ = 0.021, *P*_Transfer events_ = 0.007, *n* = 5) (Fig. 6D).

We next tested the expectation that the spatial intermixing of the plasmid-carrying and -free cells within the plasmid donor cell row along the collision boundary will affect the spatial intermixing of transconjugants (blue) and plasmid-free (gray) cells in the potential recipient cell row. Upon collision, the spatial distribution of plasmid-carrying cells in the donor colony determines the spatial distribution of plasmid-carrying cells in the recipient colony, modulating the efficiency of plasmid transfer within the recipient colony (Fig. 6C). Here, we allowed plasmid transfer to occur between the plasmid donor and potential recipient cell rows and within the potential recipient cell row, but not within the plasmid donor cell row. This allowed us to isolate the effect of intermixing within the recipient colony. For collisions where plasmid-carrying cells (magenta) are highly intermixed with plasmid-free cells (green), we again found that both the total number of new transconjugants (blue) and the number of plasmid transfer events between cell rows are significantly higher (two-sample two-sided Welch test; *P* < 0.001, *P* = 0.001, *n* = 5) (Fig. 6E). We then quantified the total number of plasmid transfer events that occurred within the potential recipient cell row and found that there were significantly more events for high spatial intermixing (138 ± 21) compared to low spatial intermixing (91 ± 28) (two-sample two-sided Welch test; *P* = 0.018, *n* = 5). Indeed, over one-third of plasmid spread within the potential recipient cell row was due to plasmid transfer between cells of the same cell type (i.e., secondary plasmid transfer, 33.7% ± 5.1%).

## DISCUSSION

Understanding the determinants of plasmid transfer both within and between surface-associated biomass patches is important for predicting the spread of antibiotic resistance and other plasmid-encoded traits, especially in environments where biomass is sparsely distributed. In this study, we showed that plasmid-encoded antibiotic resistance can readily transfer between spatially discrete bacterial colonies during their growth and physical collision with each other (Fig. 1C). Colonies initially growing at longer distances from each other allow for the process of plasmid loss to purge plasmids from the colony periphery, reducing plasmid spread into adjacent colonies (Fig. 2). By expanding the parameter space, our individual-based modeling simulations demonstrate that, within the scope of this study, interactions between intrinsic properties of the plasmid-host system (plasmid transfer and loss probabilities, Fig. 3) and spatial factors (distances between inocula, Fig. 4; local population intermixing, Fig. 6) are important drivers of plasmid spread.

The importance of the plasmid transfer and loss probabilities in determining plasmid spread is contingent on the spatial distances between colonies. In the absence of antibiotic selection, there is a period during which plasmid loss upon cell division and selection for plasmid-free cells decreases the opportunities for inter-colony plasmid transfer. Both in our experiments and simulations, this is evident as a decaying relationship between the extent of new transconjugants in the recipient colony and the initial distance between the inocula. This is consistent across simulation replicates, which intrinsically display high variability due to the stochasticity of self-organizing processes occurring during colony growth (Fig. S10 and S11). Our analyses specifically determined that even if there are high plasmid transfer probabilities during this pre-collision period, plasmid loss probabilities can partially offset these effects and become a sizable determinant of plasmid spread into adjacent colonies (Fig. 5). The extent of additional plasmid transfer within the recipient colony thus depends on the interaction between the initial distance and plasmid loss probability, as closer distances allow for more transfer as long as plasmids have not been rapidly purged from the system. The segregation control system of a particular plasmid (50) and its post-segregational regulation through, for example, toxin-antitoxin systems (51) will have a large impact on its temporal persistence (52, 53), while the host-specific cost of the plasmid and mechanisms of plasmid maintenance will also determine how rapidly plasmid-free cells dominate the biomass periphery due to their higher intrinsic growth rate (54). There are many cases in which plasmids can persist within the plasmid donor population even in the absence of positive selection due to compensatory mutations that reduce or eliminate the cost of plasmid carriage (17, 55), high conjugation rates (17), or phage predation (56). In such situations, the proliferation of plasmid-free segregants would be substantially reduced, and we would therefore expect inter-colony transfer to be independent of the initial distance between the colonies, as we would not expect a reduction in plasmid load to occur prior to collision. This means that the taxonomic or genotypic composition of a colony and the biology of the antibiotic resistance-encoding plasmid will generally have large impacts on plasmid spread between adjacent colonies (57).

We found that the spatial intermixing of plasmid-carrying and -free cells along the periphery of a plasmid donor colony affects both the total plasmid load and the degree of intermixing of the plasmid-free and -carrying cells in a potential recipient colony. There is ample evidence that more intermixed populations lead to a larger number of cell-cell contacts between distinct cell types (in this case plasmid-free and -carrying types) (e.g., references 19, 58). This has, for example, consequences for the structuring of communities via metabolic exchange (59) or contact-dependent killing (60). For a contact-dependent process, such as plasmid conjugation, this leads to a larger number of possible non-redundant plasmid transfer events (i.e., transfer events from plasmid donor to potential recipient cells as opposed to transfer events from plasmid donor to adjacent plasmid donors) (61), which increases plasmid spread (20). This explains why we observed a linear increase in the number of new transconjugants upon colony collision but a decelerating increase in the number of primary plasmid transfer events between colonies (Fig. 3E and J). After all the potential recipient cells along the collision boundary receive the plasmid, there are no more plasmid-free cells for additional inter-colony plasmid transfer to occur. In our system, the plasmid kept spreading into the recipient colony due to secondary transfer, which is higher in conditions of high spatial intermixing between plasmid-carrying and -free cells.

In light of our findings, we expect that processes leading to higher spatial intermixing or closer spatial proximity between populations will increase plasmid spread. For example, in the mouse gut, the spread of antibiotic resistance-encoding plasmids increases with frequent contact between persisting plasmid donors and invading plasmid-free enteric pathogens (62, 63). Frequent physical disturbances increase the number of new donor-recipient contacts by reshuffling the spatial positioning of cells and consequently promoting more extensive plasmid transfer (21). Surface evaporation can also increase the intermixing between plasmid-carrying and -free cells and promote plasmid spread (64, 65). Upon physical contact between colonies, however, we confirmed previous experimental and theoretical work that transfer is more likely to occur at the biomass periphery (18), as shown by the narrow extent of the new *E. coli* transconjugants along the collision boundary. The model by Lagido et al. (33) established that colony collision enables conjugative transfer on surfaces, with initial spatial separation between inocula determining final transconjugant numbers. Their model, however, assumed for simplicity that upon colony contact, all recipient cells instantly become transconjugants, an assumption that effectively treats plasmid transfer as spatially homogeneous along the collision boundary. We show that transfer is restricted to the narrow collision interface but critically depends on the spatial intermixing of donor and recipient cells along the collision boundary. Our findings therefore expand the scale at which bacterial interaction neighborhoods are assumed to operate, connecting the processes driven by short-range interactions (66, 67) with those occurring between entire colonies when they expand and collide. This multi-scale perspective aligns with recent calls to connect microscale interaction processes to macroscale spatial organization in microbial ecosystems (68).

The link between plasmid dynamics within and between biomass patches sets a basis for understanding antibiotic resistance spread at the metacommunity level, which better resembles processes occurring in sparse biofilms, such as on soil particle aggregates (26) or surfaces in wastewater treatment processes (41). In addition to the processes of plasmid transfer and loss, the persistence of antibiotic resistance in microbial communities in the absence of antibiotic pressure could be explained by spatial factors, such as the spatial intermixing between cells that operate both within and between biomass patches. In spatially structured systems, such as dental plaque or soils, this conceptualization might be of interest because the spread of plasmid-encoded antibiotic resistance could be simulated based on pre-existing models from metacommunity theory (69–71). In fact, a recent study modeled plasmid dynamics between biomass patches in simulated metacommunities, finding that costly plasmids are better maintained in the system when patches are unevenly distributed (72). This allows patches to grow in density and act as plasmid reservoirs for additional transfer upon contact with other patches. This is akin to source-sink dynamics, which are a way by which a stable plasmid donor ensures the plasmid is maintained in unstable plasmid recipients by frequent transfer (16). Our study expands such findings by showing that the physical proximity between sources and sinks determines plasmid spread between biomass patches, and that consideration of the spatial intermixing of plasmid-carrying cells within patches is a yet unrecognized factor driving plasmid spread. By combining our predictive framework with a metacommunity model that explicitly considers the spatial distribution of biomass patches, it becomes increasingly feasible to establish predictions of plasmid fate in sparse biofilms.

As shown by our experiments and simulations, the spatial distribution of plasmid-carrying and -free cells changes over time due to the de-mixing of populations during surface growth. This is the result of stochastic fluctuations in the boundaries between growing populations that result in strong genetic drift (22, 73). This implies that the degree of intermixing between populations is a time-dependent process, such that collisions occurring before de-mixing are predicted to increase plasmid spread. Previous work has shown that the timing of antibiotic administration in surface-associated bacterial systems can determine plasmid spread by creating a positive selective pressure when plasmids are still readily transferring within the community due to high intermixing (74). As intermixing decreases over time, the spread of plasmid-mediated antibiotic resistance becomes increasingly confined to fewer regions where plasmid-carrying and -free cells are still in contact. Considering the temporal dynamics of the population de-mixing process is therefore important for predicting plasmid spread into neighboring biomass.

We acknowledge that the relative simplicity of our experimental system and modeling approach has several limitations. Our simplified consortia can effectively minimize confounding factors and enable us to draw strong conclusions, but this comes with the cost of not being able to capture all the complex interactions and processes within collections of colonies, such as in sparse multi-species biofilms. We have not tested the dependency of plasmid transfer on the bacterial composition of the adjacent colliding colonies. Some bacteria can produce a thick layer of exopolysaccharides that can prevent cell-cell contacts even when there are compressive physical forces at play (75). Also, some bacteria might not have compatible conjugation machineries to establish the pili junctions required for effective transfer (76), while some biofilms will prevent collisions via chemical signaling (77). The generalizability of our findings could be confirmed with studies that implement our simplified experimental system using diverse sets of strains from multiple taxonomic groups with varying traits. There are also multiple factors we could not address that will determine the extent of this spread into recipient biomass. First, physical forces might push new transconjugants toward the collision boundary, uplifting them and creating a vertical rather than a horizontal expansion (70). Second, the metabolic state of cells closer to the center of the biomass can determine their ability to capture and transfer the plasmid. Here, nutrient availability, interspecies interactions, and abiotic stressors might play an important role in determining such cellular activity. Finally, plasmid loads vary depending on the metabolic costs that plasmids impose on host cells (47) and on the selective pressures acting on plasmid-encoded traits (48), making it necessary in future studies to include plasmid costs and selective pressures as factors of antibiotic resistance spread in spatially explicit systems.

### Conclusions

Our study reveals how the interplay between plasmid dynamics and microbial spatial organization determines the spread of plasmid-encoded antibiotic resistance, offering opportunities to anticipate and manage this global challenge. By linking plasmid transfer and loss probabilities to spatial factors, such as physical distances between microbial colonies and intermixing between distinct populations, we establish quantitative relationships that can be integrated into predictive models of resistance dissemination across natural and clinical environments. These insights can facilitate the design of strategies that limit resistance spread on surfaces (78), for example, via surface engineering (79), targeted treatment of discrete plasmid-carrying populations (80), or accounting for the 3D structure of biofilms (81). Ultimately, our framework moves toward a more mechanistic and predictive understanding of when and where antibiotic resistance will persist in spatially structured microbial ecosystems.

## MATERIALS AND METHODS

### Bacterial strains and plasmid

The plasmid donor colonies consisted of two genetically engineered isogenic mutants of the bacterium *Stutzerimonas stutzeri* A1601, whose genetic modifications and growth traits were established previously (82, 83). Each strain is genetically identical to the other except for carrying a different isopropyl β-D-1-thiogalactopyranoside (IPTG)-inducible fluorescent protein-encoding gene located on the chromosome (*egfp* encoding for green fluorescent protein or *echerry* encoding for red fluorescent protein) (Table S5). This enables us to distinguish and quantify the different strains when grown together in a spatially explicit manner. We transformed the strain carrying *echerry* with the low copy number plasmid pAR145 (pSU2007 aph::cat-PA1/04/03-cfp*-T0, described in reference 84). pAR145 is a derivative of plasmid R388 and encodes for chloramphenicol resistance and an IPTG-inducible *ecfp* gene (encoding for cyan fluorescent protein). The potential recipient strain was *E. coli* DH5α [F− supE44 lacU169 (ϕ80lacZΔM15) hsdR17 recA1 endA1 gyrA96 thi-1 relA1] (85), which was grown in lysogeny broth (LB) overnight at 30°C.

### Quantification of plasmid cost

We used a previously described colony collision assay (86) to measure the cost of carrying pAR145 in terms of its effect on growth rate in the absence of positive selection. We grew the pAR145-carrying and -free *S. stutzeri* strains in isolation and adjusted the OD600 of each overnight culture to 2.0 with 0.89% (wt/vol) sodium chloride solution. We then placed a 1 µL drop of the pAR145 donor mixture and another 1 µL drop of the potential recipient 3 mm apart from each other on replicated LB agar plates (1.5% agar). We incubated the plates at room temperature for 96 h, which allowed the colonies to grow and eventually collide into each other. We estimated the relative growth rates of the pAR145 donor and potential recipient strains using the curvature of the arc of the collision boundary between the colonies and the radii of the colonies using the following equation:


$$R_{b}=l\frac{v_{1}v_{2}}{\left|v_{1}^{2}-v_{2}^{2}\right|}=l\frac{1+s}{s(2+s)},$$

where *l* is the distance between the centroids of two colonies, *s* is the cost of carrying pAR145, *v*_1_ is the expansion velocity of the pAR145-free colony, and *v*_2_ is the expansion velocity of the pAR145-carrying colony. *R_b_* is the radius of the circle generated by the arc at the collision boundary, which together with the mentioned parameters, allows the derivation of *s*. We quantified *R_b_* and *l* for four biological replicates using Adobe Illustrator (v27.0.1). See Table S1 for detailed data. These calculations allowed us to set realistic parameters for our individual-based modeling (see below).

### Colony collision experiments

We performed colony collision experiments where we inoculated a mixture of the *S. stutzeri* strains (the plasmid donor colony) and *E. coli* DH5α (the potential recipient colony) as single droplets onto replicated LB agar plates and allowed them to grow until they physically collided with each other. We initially grew all the strains separately in oxic liquid LB medium overnight at 37°C and set their optical densities at 600 nm (OD600) to 2. We next mixed the two *S*. *stutzeri* strains (plasmid-carrying and -free) at a 1:1 ratio (vol:vol). Using an Evo 200 robotic liquid handling system (Tecan, Männedorf, Switzerland), we deposited pairs of droplets of the *S. stutzeri* mixture and the *E. coli* strain (1 µL of each culture) at four discrete spatial positions on each LB agar plate adjusted to a pH of 7.5 and amended with 1 mM IPTG. We programmed the liquid handling system to deposit the *S. stutzeri* mixture and *E. coli* droplets at distances between droplet centroids of 2.80, 2.90, 3.00, 3.10, 3.20, 3.30, 3.40, 3.50, 3.70, and 4.00 mm with three replicates per distance. We incubated the plates for 96 h at room temperature in oxic conditions. These experiments allowed us to quantify the transfer of pAR145 from plasmid-carrying cells in the *S. stutzeri* donor colony (visualized as cyan or magenta) into plasmid-free cells in the *E. coli* recipient colony (visualized as blue cells).

### Bacterial colony imaging and quantitative image analysis

We imaged the colonies immediately after completion of the incubation period using a Leica TC5 SP5 II confocal microscope (Leica Microsystems, Wetzlar, Germany) with objectives 10×/0.3 NA (dry) and 63×/1.4 NA. We scanned the entire colony areas (*S. stutzeri* mixture and *E. coli*) by stitching together multiple frames of 1,024 × 1,024 pixels. We set the laser emission to 514 nm for the excitation of red fluorescent protein, 488 nm for the excitation of green fluorescent protein, and 458 nm for the excitation of cyan fluorescent protein. We analyzed the images in ImageJ (https://imagej.nih.gov/ij/) using FIJI plugins (v.2.1.0/1.53c; https://fiji.sc).

We quantified plasmid load (i.e., the total area of cyan fluorescent protein-expressing cells) using the “Sholl analysis” plugin (87) on the binarized image of the cyan channel after application of a noise-reduction threshold of 20 pixels. The “Sholl analysis” calculated the number of cyan pixels at 10 µm radial increments from the centroid to the colony edge. We initiated the analysis at 1,500 µm from the colony centroid because this distance represents the edge of the inoculation droplet. We then applied the “Area to line” function of the “Overlay” plugin to register the number of cyan pixels for the areas corresponding to each 10 µm radial increment. We defined plasmid load as the total number of cyan pixels divided by the total number of pixels along each radius.

### Individual-based modeling of colony collisions

We performed individual-based modeling of colony growth and collision using CellModeller 4.3, a Python-based computational framework designed to model the growth and division of multicellular systems (49). We ran all simulations on the ETH Euler high-performance computing cluster using the Slurm workload manager for batch job submissions. We modeled individual bacteria as three-dimensional rod-shaped capsules using the CLBacterium biophysical module with growth restricted to a monolayer. We initialized cells to have a length of 3.5 units and allowed them to elongate along their long axis at a rate set by their growth rate parameter, with a growth drag coefficient gamma = 20. For each newly born cell, we defined an individual division threshold by adding a Gaussian-distributed increment (mean 2.0 and standard deviation 0.45) to its length at birth. Cells divided when their size exceeded this threshold, producing two daughter cells of approximately half the parent cell’s division length and each assigned a new division threshold. This constant added-size (length) can accurately model bacterial division and cell size homeostasis (88). We ran each simulation until reaching a total population size of 20,000 cells.

To study plasmid dynamics during colony expansion and collision, we simulated two spatially separated colonies composed of cell types with distinct backgrounds, which we refer to as the plasmid donor and potential recipient colonies. We then allowed them to grow and physically collide into each other. We encoded each combination of colony background (plasmid donor or potential recipient) and plasmid state (plasmid-free or -carrying) as a discrete cell type and updated these labels whenever plasmid transfer or loss occurred. For the simulations reported in Fig. 3 to 5, we initialized both colonies as circles (radius 40 units) with their centers d units apart (*d* = 80, 90, 100, 110, 120, 130). These distances are equivalent to cell lengths *d* = 23, 26, 29, 32, 35, 38, respectively. The donor colony contained 200 cells placed at random positions within the circle in a 1:1 mixture of plasmid-free (green) and -carrying (magenta) cells. The potential recipient colony contained 200 plasmid-free cells (gray) placed at random positions within the second circle on the same *xy*-plane. We set the growth rate = 1.0 for plasmid-free cells in the plasmid donor colony, 0.83 for plasmid-carrying cells in the plasmid donor colony, and 0.8 for both the plasmid-free and -carrying cells in the potential recipient colony, matching our experimentally measured plasmid costs (reported in reference 17). Thus, plasmid carriage imposed a 17% growth-rate cost in the plasmid donors but no additional cost in the potential recipient cells.

We extended the model to incorporate plasmid transfer and loss. We simulated contact-dependent plasmid transfer using the neighbor lists returned from the CLBacterium (set by parameter “compNeighbours = True”) (20). At each time step, a plasmid-free cell in contact with a plasmid-carrying cell switches to the corresponding plasmid-carrying cell type with a defined probability (referred to as the plasmid transfer probability). We simulated plasmid loss during cell division. At each cell division, one of the two daughter cells is chosen at random and switches to a plasmid-free cell of the same cell type with a defined probability (referred to as the plasmid loss probability). We assumed that plasmid-carrying cells in the potential recipient colony were stable (plasmid loss probability = 0), as we observed experimentally. We tracked all plasmid transfer and loss events using dedicated counters throughout each simulation. We performed 1,500 unique simulations using the following full factorial design: plasmid transfer probability (5 levels: 0, 0.001, 0.002, 0.003, 0.004) × plasmid loss probability (5 levels: 0, 0.25, 0.5, 0.75, 1.0) × distance between inocula (6 levels: 80, 90, 100, 110, 120, 130) × 10 replicates.

To test the effect of spatial intermixing of plasmid-donor and -free cells on plasmid spread, we placed cells from the plasmid donor and potential recipient cell types along two parallel cell rows. These cell rows were placed immediately contiguous to each other and with cells allowed to grow in all directions, such that plasmids from the donor cells could be readily transferred to the recipient cells. The plasmid donor row contained a 1:1 mixture of plasmid-carrying and -free cells while the potential recipient row contained only plasmid-free cells. We assigned the initial rotational orientation of each cell at random as in the previous simulations. In this setup, we considered two initial configurations for the donor row: a pattern with high spatial intermixing (alternation of plasmid-carrying and plasmid-free cells) and a pattern with low spatial intermixing (spatial segregation by type). We set the growth rate of all cell types to be the same because the length and time scales are small when simulating collision boundaries and to isolate the effects of spatial positioning from differences in growth rates. We used the same values for all other settings used for the simulations, except that we removed the plasmid loss process for this short time scale. Because the initial cell rows collided nearly immediately after the start of the simulation, most growth occurred in the radial direction. The consequence is that the final simulation images appear circular with two clear hemispheres on each side of the original cell rows.

### Statistical analysis

We used the Shapiro-Wilk test to test for non-normality of the data and the Breusch-Pagan test to identify heteroscedasticity. Both tests resulted positive in most simulation data, we used non-parametric statistics unless otherwise stated. Specifically, we used Spearman’s rank correlations to test for monotonic relationships between variables of plasmid spread—number of transconjugants and primary plasmid transfer events—and our explanatory variables. We then tested for treatment effects and interactions between factors using GLMs fitted with a negative binomial distribution using the MASS R package (v7.3-60). We used the likelihood ratio test to test for main effects of the explanatory variables. When we lacked sufficient data to test whether our data sets deviate from normality, we performed treatment comparisons using two-sample two-sided Welch tests, which accommodate unequal variance between groups. We performed all statistics in R (v4.4.1 [89]).

## Supplementary Material

Reviewer comments

## Data Availability

All data and code generated in this study have been deposited in the Eawag Research Data Institutional Repository (https://opendata.eawag.ch/) and are publicly available at https://doi.org/10.25678/000H4Y.

## SUPPLEMENTAL MATERIAL

Supplemental figures and tables msystems.00531-26-s0001.docx Tables S1 to S5 and Figures S1 to S14.

OPEN PEER REVIEW reviewer-comments.pdf An accounting of the reviewer comments and feedback.

ASM does not own the copyrights to Supplemental Material that may be linked to, or accessed through, an article. The authors have granted ASM a non-exclusive, world-wide license to publish the Supplemental Material files. Please contact the corresponding author directly for reuse.
