# Supplementary material for: Spatial constraints determine the spread of plasmid-encoded antibiotic resistance between bacterial colonies
Source: mSystems. 2026 Aug 28;11(9):e00531-26. doi: 10.1128/msystems.00531-26 (PMC13595988; doi:10.1128/msystems.00531-26)
Supplement: Supplementary file 1 [file reviewer-comments.pdf]

ASM | mSystems

Spatial constraints determine the spread of plasmid-encoded antibiotic resistance between bacterial colonies

Josep Ramoneda, Deepthi Vinod, Yinyin Ma, Chujin Ruan, Julian Schmidt, Michael Manhart, Daniel Angst, and David Johnson

Corresponding Author(s): Josep Ramoneda, Centre d'Estudis Avancats de Blanes

Review Timeline:

Submission Date:	April 24, 2026
Editorial Decision:	June 1, 2026
Revision Received:	July 6, 2026
Accepted:	August 6, 2026

Editor: William Harcombe

Reviewer(s): Disclosure of reviewer identity is with reference to reviewer comments included in decision letter(s). The following individuals involved in review of your submission have agreed to reveal their identity: Kaitlin Schaal (Reviewer #1)

Transaction Report:

(Note: With the exception of the correction of typographical or spelling errors that could be a source of ambiguity, letters and reports are not edited. The original formatting of letters and referee reports may not be reflected in this compilation.)

DOI: https://doi.org/10.1128/msystems.00531-26

Re: mSystems00531-26 (**Spatial constraints determine the spread of plasmid-encoded antibiotic resistance between bacterial colonies**)

Dear Dr. Josep Ramoneda:

Thank you for the privilege of reviewing your work. Below you will find my comments, instructions from the mSystems editorial office, and the reviewer comments.

There was broad enthusiasm for the rigor of experiments and models used to quantify factors determining plasmid transmission between colonies and consensus that this work has value for the field. However, both reviewers highlight aspects of the manuscript they found unclear. I encourage the authors to consider methods of improving the clarity of the methods, rationale and implications of the work.

Please return the manuscript within 60 days; if you cannot complete the modification within this time period, please contact me. If you do not wish to modify the manuscript and prefer to submit it to another journal, notify me immediately so that the manuscript may be formally withdrawn from consideration by mSystems.

Revision Guidelines
To submit your modified manuscript, log into the submission site at https://msystems.msubmit.net/cgi-bin/main.plex. Go to Author Tasks and click the appropriate manuscript title to begin. The information you entered when you first submitted the paper will be displayed; update this as necessary. Note the following requirements:

• Upload point-by-point responses to the issues raised by the reviewers in a file named "Response to Reviewers," NOT in your cover letter.
• Upload a compare copy of the manuscript (without figures) as a "Marked-Up Manuscript" file.
• Upload a clean .DOC/.DOCX version of the revised manuscript and remove the previous version.
• Each figure must be uploaded as a separate, editable, high-resolution file (TIFF or EPS preferred), and any multipanel figures must be assembled into one file.
• Any supplemental material intended for posting by ASM should be uploaded with their legends separate from the main manuscript. You can combine all supplemental material into one file (preferred) or split it into a maximum of 10 files with all associated legends included.

For complete guidelines on revision requirements, see our Submission and Review Process webpage. Submission of a paper that does not conform to guidelines may delay acceptance of your manuscript.

Data availability: ASM policy requires that data be available to the public upon online posting of the article, so please verify all links to sequence records, if present, and make sure that each number retrieves the full record of the data. If a new accession number is not linked or a link is broken, provide mSystems production staff with the correct URL for the record. If the accession numbers for new data are not publicly accessible before the expected online posting of the article, publication may be delayed; please contact production staff (mSystems@asmusa.org) immediately with the expected release date.

Publication Fees: For information on publication fees and which article types are subject to charges, visit our website. If your manuscript is accepted for publication and any fees apply, you will be contacted separately about payment during the production process; please follow the instructions in that e-mail. Arrangements for payment must be made before your article is published.

ASM Membership: Corresponding authors may join or renew ASM membership to obtain discounts on publication fees. Need to upgrade your membership level? Please contact Customer Service at Service@asmusa.org.

The ASM Journals program strives for constant improvement in our submission and publication process. Please tell us how we can improve your experience by taking this quick Author Survey.

Thank you for submitting your paper to mSystems.

Sincerely,
William Harcombe
Editor
mSystems

Reviewer #1 (Comments for the Author):

Overall, a very cool and exciting piece of research. Detailed comments in the attachment.

Reviewer #2 (Comments for the Author):

The manuscript "Spatial constraints determine the spread of plasmid-encoded antibiotic resistance between bacterial colonies" explores the influence of plasmid transfer and loss in the spread of antibiotic resistance on surfaces, particularly concerning the distance between donor and recipient populations. By combining structured colony experiments with an individual-based modelling approach, the study shows how important spatial organization is for plasmid conjugation and antimicrobial resistance dissemination.

Overall, this study offers a useful quantitative analysis that connects plasmid transfer, plasmid loss, and the spatial structure of bacterial colonies. The integration of experiments and individual-based modelling is coherent and well executed, and it refines current understanding of how spatial organization shapes the spread of antibiotic resistance. The study also addresses its limitations, such as cell type, compatibility for conjugation, and simplifications in the model.

The impact and general applicability of this study could increase by broadening the modelling framework beyond its current parameters. Specifically, finding critical thresholds for crucial factors like plasmid cost, transfer probability, temporality, and spatial separation would allow for a broader assessment of conjugation limits. Such analysis (already sketched in Fig. 5) would make this work more relevant as a widely applicable predictive tool.

Furthermore, it would be beneficial to discuss in depth the model predictions shown in Fig. 6. The spatial organization of the donor colony shown in Fig. 1 suggests that the system may not be accurately described by a single static mixing pattern throughout colony development. The donor colony's periphery shows a low-intermixing structure, with plasmid-bearing cells being located in relatively distant sectors, while the inner region of the colony shows a higher degree of intermixing and more plasmid carriers. This observation indicates that conjugation dynamics could vary over time during colony collision, reflecting changes in spatial structure as the colony grows and matures. It would be interesting to investigate whether including the temporal evolution of spatial organization in the modelling during colony collision could capture potential shifts in conjugation dynamics. An extensive discussion on how low- and high-intermixing regimes develop and how timing affects conjugation dynamics would enhance the understanding of the observed spatial dynamics.

The authors use an empirical fluorescent bacteria and plasmid system together with an individual based model to investigate plasmid transfer between bacterial colonies. They show that plasmid transfer into the recipient colony decreases as distance between colonies increases because the plasmid is increasingly lost from the donor cells at the expanding colony edge. The authors further investigate the effect of varying plasmid transfer and loss rates on these outcomes, as well as what happens when, in a donor colony with both plasmid-free and plasmid-carrying cells, plasmid-carrying cells are grouped together or interspersed with plasmid-free cells.

The work is both technically sound and an exciting and valuable set of results regarding plasmid dynamics and that contributes to thinking about microbiomes as metacommunities and about plasmid spread. The work should be better contextualized/situated within microbiome ecology, and some parts of the work should be more clearly explained, and decisions motivated.

Major comments

Figure 6:

There was quite a bit that I didn't understand regarding Figure 6 and the associated results and methods sections. I struggled to visualize what was meant in the description of the methods (lines 628-639), and between this, the results section, and the figure legend, I don't understand how the circular colonies were generated or how many steps were in the process. For example, why does it matter that cells were rotated randomly (lines 632 and 975-6)?

I recommend not calling it a cell wall – this is part of what confused me. I would call it a line or row of cells, and call the groups of cells line segments rather than patches. How do the two lines of cells turn into one circular colony? At the start of the simulation, are they directly adjacent, or to the either move or replicate/grow toward each other? Line 338 says that the cell walls collide and then a circular colony forms, but I struggle to visualize the process this is meant to describe. Lines 339-40 say that the "left and right hemispheres contain information regarding plasmid spread from the plasmid donor to the plasmid recipient cell wall" – I am not sure what this means. Lines 361-2 ("spatial features of the plasmid donor hemisphere are acquired by the potential recipient hemisphere upon collision") I find similarly obscure.

Panels B and C each show groups of three lines of cells and a circular colony, grouped by "low intermixing" and "high intermixing." The description in the legend (lines 976-9) isn't detailed enough for me to understand what is shown. How do B and C differ? This is answered in the text at lines 348-9 & 366, but is very difficult to interpret from the figure/legend as they are currently, which also hinders comparison of panels D and E. Additionally, I struggle to interpret the relationship among the three grouped lines and their meaning. I think they are showing sequential modeling steps first of intra-line HGT and then between-line HGT?

Why did you hypothesize that spatial organization would be a determinant of plasmid spread (lines 324-5)? Why did you decide to test a case where there was no HGT among recipient cells and a case with no HGT among donor cells? How do you interpret the role of increased intermixing in light of these restrictions?

Ecological context

The introduction and discussion position the work as related to clinical microbiology (e.g. surface-associated microbiomes, examples referenced in line 116) and natural microbiomes

(e.g. examples referenced in lines 453-4), but this positioning is sparse. By making more frequent and more explicit connections to both clinical and natural microbial ecology, the work can be presented in a more impactful way. For example, in line 105 when surfaces are mentioned. In line 116, only clinical examples are mentioned.

This work seems related to existing microbial ecology research into interaction neighborhoods, which might be a nice connection to make – interaction neighborhoods across scales, considering within-colony plasmid transfer and between-colony transfer.

Minor comments

32: define sparse here, e.g. by making "composed of discrete bacterial colonies" a parenthetical.

93: "in addition to nutrient availability" due to host growth, presumably?

129: it's not yet clear why you use the pair of fluorescently labelled conspecifics – consider omitting this information here.

141: "using a full-factorial design" – it's not clear yet what all the factors are. Either state the factors, or omit this information here.

155-7: why did you first quantify plasmid dynamics within the donor colony? This is worth justifying better here, especially in the context provided, which is antibiotic treatment/resistance. Under antibiotic treatment, we would expect the plasmid to be at or near 100% prevalence within the colony.

163: intra?

185: "based on a prior study" → "based on Lagido et al.'s model"

188: we would therefore expect this time to vary across host-plasmid pairs, as it would depend on specific plasmid cost and mechanisms of plasmid maintenance. Plasmid maintenance is touched on in the discussion, but might be worth briefly mentioning these two factors here (and worth mentioning host-specific plasmid costs in the discussion).

208: might be worth listing the factors here (distance x transfer probability x loss probability)

224: what is a primary plasmid transfer event?

Supplementary tables 2-4: I can't tell from the table legends in the supplement what makes the information in these tables different. It would be nice to distinguish them more clearly in the legends so that cross-referencing from the text is easier.

Supplementary figures 4-7: label panel headers (e.g. Di = 80 units), or specify in legends

253: "a significant negative effect of the plasmid loss probability through its interaction with distance" – why would there be an interaction with distance for secondary transfer?

272-3: why would this be the case?

288-289: any idea why there would be a threshold colony size where primary transfer slows down?

308-310: is there an ecological interpretation for this?

324-5: why did you hypothesize this? Again, it's worth providing more motivation here.

330-1: just to check that I've understood correctly – in this study, "intra-colony" and "secondary" mean the same thing regarding plasmid transfer, and likewise "inter-colony" and "primary"? Similarly, the process described in lines 375-7 could also be called secondary transfer?

346: "by modulating the efficacy of interspecific plasmid transfer" → by increasing the number of plasmid-carrying donor cells

375-7: This would be secondary transfer?

391: how can you say that plasmid transfer, plasmid loss, and colony distance (the three factors you tested) are the primary drivers of plasmid spread when you didn't test other factors? Do you mean that within the scope of the study they are all relevant to plasmid spread (in which case the current statement is misleading)?

406-9: it's worth mentioning segregation control, eg. toxin-antitoxin systems, here when talking about mechanisms that maintain plasmids in the absence of environmental selection for their genetic cargo.

424-30: can you explicitly bridge this gap? Presumably the transconjugants not accounted for due to primary transfer are due to secondary transfer, indicating that the plasmid is continuing to spread spatially within the recipient colony.

446-7: "critically depends…" I assume this is in reference to the Figure 6 results, and the "foldedness" of the border observed in the round colonies in panels B and C. Do you know if the borders of high intermixing simulations are longer than the low intermixing borders due to this increased two-dimensional surface area? Also, in the text here do you refer to intermixing of the two types of donor cells, or intermixing between donor and recipient cells?

459-60: why would you expect spatial factors to be a more important driver in natural microbiomes than plasmid transfer and loss rates?

468: "even when the plasmid is unstable in all community members" Where is this shown? Is this referring to plasmid loss rates tested (which figure)? More clarification is needed here. This also differs from the experimental source-sink results, where the plasmid is stable in the source host.

468-71: what makes these results different and interesting compared to plasmid research that focuses on conjugation/transfer rates isn't coming through enough here. If you can make this clear, it will increase the impact of the work.

491-4: did you test this here? Is this referring to existing work (citation(s))? You haven't made the link to plasmid cost clear enough here, nor the link to the work presented here.

524: please include the pure culture growth conditions (media and temperature).

652: suggestion to include citations for software and packages

Figure 1:

Please explain more clearly how to interpret the fluorescence that we see. In the images, I see red, green, purple, and blue.
In panel C, how did you distinguish the types of HGT indicated by the different arrows? Please explain in the methods as well.

Figure 2:
Can something be done to increase the visibility of the blue patches in panel A?
For the overlapping dots in panel B, try reducing the alpha so that layered points show up darker.
Out of curiosity and ecological interest, can you explain what is happening at the collision edge to give it the arc shape? Is the *Pseudomonas* colony swarming/replicating around the edge of the *E. coli* colony, or is the *E. coli* colony replicating into the *Pseudomonas* colony?

Figure 4:
In Panel A it says D, not Di.
At lines 949 & 952, "(blue in A)".
At lines 949 & 951, "initial distance (Di)".
Line 954: the colors are from B & C

Figures S13 and S14: suggestion to refer to them in the results and give them a little interpretation rather than only in the discussion. Also, the A and B are difficult to see against the dark purple.

Response to Reviewers

Dear Dr. Harcombe, Editor of mSystems,

See below the point-by-point responses to the comments and suggestions raised by reviewers, which we thank for their constructive feedback.

The main aspects that required revision were unclarity in the explanation of the approach and results presented in Figure 6, which show the importance of spatial intermixing of populations on plasmid spread, whose description we now developed both in the methods, results, and discussion sections. We have also expanded discussion on this topic and the importance of temporal changes to the spatial positionings of populations on plasmid spread by adding an entire paragraph in the discussion. Additional important comments had to do with providing more motivation for the hypotheses and analyses presented, which we have solved by adding relevant references and additional description, as we realized this was necessary to readers unfamiliar with this specific research topic.

Reviewer #1 (Comments for the Author):

Overall, a very cool and exciting piece of research. Detailed comments in the attachment.

The authors use an empirical fluorescent bacteria and plasmid system together with an individual based model to investigate plasmid transfer between bacterial colonies. They show that plasmid transfer into the recipient colony decreases as distance between colonies increases because the plasmid is increasingly lost from the donor cells at the expanding colony edge. The authors further investigate the effect of varying plasmid transfer and loss rates on these outcomes, as well as what happens when, in a donor colony with both plasmid-free and plasmid-carrying cells, plasmid-carrying cells are grouped together or interspersed with plasmid-free cells.

The work is both technically sound and an exciting and valuable set of results regarding plasmid dynamics and that contributes to thinking about microbiomes as metacommunities and about plasmid spread. The work should be better contextualized/situated within microbiome ecology, and some parts of the work should be more clearly explained, and decisions motivated.

We really appreciate the constructive feedback and have implemented your suggestions to improve the clarity and general interest of the work.

Major comments

Figure 6:
There was quite a bit that I didn’t understand regarding Figure 6 and the associated results and methods sections. I struggled to visualize what was meant in the description of the methods (lines 628-639), and between this, the results section, and the figure legend, I don’t understand how the circular colonies were generated or how many steps were in the process. For example, why does it matter that cells were rotated randomly (lines 632 and 975-6)?

We apologize for the lack of clarity in this presentation. We have now improved the description of the methods used to generate simulations in this figure and include a more detailed explanation of the results. The random rotation of cells was as in the other simulations and,

because it caused confusion, we now keep this information only in the methods section. As explained in L677-692 (Methods section), the cell rows were placed in parallel and contiguous to each other, with cells allowed to grow in all directions, which results in an initial collision with plasmid transfer from donor to recipient cells, and then radial growth that resembles that of the other simulations where cells were initially placed at the center. That is what creates the circularity of the colonies, but with the benefit that each hemisphere contains the properties (i.e. distribution of plasmid-carrying cells) acquired when the cell rows interact. We now clarify this in the following lines.

> **Lines 673-681:** "…we placed cells from the plasmid donor and potential recipient cell-types along two parallel cell rows. These cell rows were placed immediately contiguous to each other and with cells allowed to grow in all directions, such that plasmids from the donor cells could be readily transferred to the recipient cells. The plasmid donor row contained a 1:1 mixture of plasmid-carrying and -free cells while the potential recipient row contained only plasmid-free cells. We assigned the initial rotational orientation of each cell at random as in the previous simulations. In this setup, we considered two initial configurations for the donor row: a pattern with high spatial intermixing (alternation of plasmid-carrying and plasmid-free cells) and a pattern with low spatial intermixing (spatial segregation by type)".

> **Lines 686-689**: "Because the initial cell rows collided nearly immediately after the start of the simulation, most growth occurred in the radial direction. The consequence is that the final simulation images appear circular with two clear hemispheres at each side of the original cell rows".

I recommend not calling it a cell wall – this is part of what confused me. I would call it a line or row of cells, and call the groups of cells line segments rather than patches. How do the two lines of cells turn into one circular colony?

Changed as recommended. In addition, see our response and explanations above for how/why they become circular.

At the start of the simulation, are they directly adjacent, or to the either move or replicate/grow toward each other? Line 338 says that the cell walls collide and then a circular colony forms, but I struggle to visualize the process this is meant to describe.

The two cell rows are adjacent to each other at the initial condition, and the cells physically collide (i.e. impose mechanical forces on each other) as they expand in all directions (as with all our other simulations). Because the cell rows are initially placed very close to each other, they quickly impose mechanical forces on each other and start to grow outwards due to the dense packing of cells at the center, consequently forming a circular colony. We now elaborate on this in the following lines of the revised manuscript.

> **Lines 346-350:** "We used our individual-based modelling framework to simulate the collision boundary as two cell rows, where the plasmid donor cell row consisted of plasmid-carrying (magenta) and -free (green) cells and the recipient cell row consisted of a different population of plasmid-free (grey) cells (Fig. 6A)".

> **Lines 353-355**: "After the cell rows collide, a single circular colony forms where the left and right hemispheres contain information regarding plasmid spread from the plasmid donor to the potential recipient cell row."

Lines 339-40 say that the "left and right hemispheres contain information regarding plasmid spread from the plasmid donor to the plasmid recipient cell wall" – I am not sure what this means. Lines 361-2 ("spatial features of the plasmid donor hemisphere are acquired by the potential recipient hemisphere upon collision") I find similarly obscure.

Both hemispheres form after the outward growth of the cell rows that have initially collided, such that plasmids that were transferred during that initial collision could spread into the recipient (grey) hemisphere. We agree the acquisition of "spatial features" is a vague term, as what we are referring to is the spatial distribution of recipient cells that acquired the plasmid. We now clarify this in the following lines of the revised manuscript.

> **Lines 379-381**: "Upon collision, the spatial distribution of plasmid-carrying cells in the donor colony determines the spatial distribution of plasmid-carrying cells in the recipient colony, modulating the efficiency of plasmid transfer within the recipient colony (Fig. 6C)."

Panels B and C each show groups of three lines of cells and a circular colony, grouped by "low intermixing" and "high intermixing." The description in the legend (lines 976-9) isn't detailed enough for me to understand what is shown. How do B and C differ? This is answered in the text at lines 348-9 & 366, but is very difficult to interpret from the figure/legend as they are currently, which also hinders comparison of panels D and E.

We agree that the caption did not clearly describe what is shown in panels B and C. The difference between these panels is that in panel B plasmid transfer is allowed within the donor cell row but not within the recipient cell row, whereas in panel C it is not allowed within the donor cell row but is allowed within the recipient cell row. This facilitates the isolation of plasmid load (which is higher when transfer is allowed within highly intermixed donor colonies), additional transfer within the recipient colony, and spatial intermixing. We have now revised the caption in Figure 6 to make this distinction clear and align it with the main text. These changes are as follows.

> **Lines 1131-1135 (Figure 6 caption)**: "(B) Simulation images where plasmid transfer was allowed within the donor cell row but not among cells in the recipient cell row. (C) Simulation images where plasmid transfer was allowed within the recipient cell row but not among cells in the donor cell row. Schematic illustrations of the expected effects are on the left and the simulation outputs are on the right in B and C."

Additionally, I struggle to interpret the relationship among the three grouped lines and their meaning. I think they are showing sequential modeling steps first of intra-line HGT and then between-line HGT?

We now clarify that both intra- and between-row HGT occurs simultaneously, not sequentially. This happens only in the simulations shown in panel B which allowed intra-line HGT. We make this clear in the following lines of the revised manuscript.

> **Lines 1135-1138 (Figure 6 caption)**: "As cell rows expand in all directions, these initially collide and plasmids transfer from the donor to recipient cell row. Growth then proceeds in a radial manner due to the dense packing of cells at the center, thus forming a circular colony with two distinct hemispheres."

Why did you hypothesize that spatial organization would be a determinant of plasmid spread (lines 324-5)?

Previous work has shown that the spatial positioning of plasmid-carrying and -free cells, particularly when they are highly intermixed, leads to more efficient plasmid spread, as this intermixing increases physical contacts between plasmid-carrying and plasmid-free cells (e.g., see Ma et al., Cell Reports). We now cite additional literature to support this hypothesis and clarify this reasoning in the following lines of the revised manuscript.

> **Lines 338-341:** "More intermixed plasmid-carrying and -free populations can enable more efficient plasmid transfer than conditions where populations are spatially segregated (19, 20), as the number of contacts between plasmid-carrying and -free cells is higher when they are intermixed."

Why did you decide to test a case where there was no HGT among recipient cells and a case with no HGT among donor cells? How do you interpret the role of increased intermixing in light of these restrictions?

In the case of no HGT among recipient cells, we wanted to isolate effects of the spatial positionings of plasmid donor cells from additional plasmid spread due to subsequent HGT within the recipient hemisphere. In the case of no HGT among donor cells, we wanted to isolate the influence of the spatial positioning of donor cells from the higher plasmid load in the recipient colony when cells are highly intermixed (we would otherwise have the same situation of the experimental system where the area with the highest plasmid load is also the area with the highest intermixing). We now clarify this in the following lines of the revised manuscript.

> **Lines 361-368**: "Higher spatial intermixing will increase the number of plasmid-carrying donor cells as well as reducing the total number of redundant plasmid transfer events (transfer occurring between donor cells and recipient cells that already have the plasmid). To test this, we simulated two scenarios to isolate the effects of spatial intermixing within the donor cell row from those of spatial intermixing in the recipient cell row. Thus, we first allowed plasmid transfer to occur within the plasmid donor cell row and from the plasmid donor to the potential recipient cell row, but not within the potential recipient cell row (Fig. 6B)."

> **Lines 379-385**: "Upon collision, the spatial distribution of plasmid-carrying cells in the donor colony determines the spatial distribution of plasmid-carrying cells in the recipient colony, modulating the efficiency of plasmid transfer within the recipient colony (Fig. 6C). Here, we allowed plasmid transfer to occur between the plasmid donor and potential recipient cell rows and within the potential recipient cell row, but not within the plasmid donor cell row. This allowed us to isolate the effect of intermixing within the recipient colony."

Ecological context
The introduction and discussion position the work as related to clinical microbiology (e.g. surface-associated microbiomes, examples referenced in line 116) and natural microbiomes (e.g. examples referenced in lines 453-4), but this positioning is sparse. By making more frequent and more explicit connections to both clinical and natural microbial ecology, the work can be presented in a more impactful way. For example, in line 105 when surfaces are mentioned. In line 116, only clinical examples are mentioned.

Thank you for noting this. We have now introduced additional references and made more explicit connections to relevant systems, now also touching upon topics such as colonization of environmental pollutants contributing to antibiotic resistance spread in the environment

such as microplastics or quantitatively important settings for antibiotic resistance spread such as wastewater treatment systems. See the following lines of the revised manuscript.

> **Lines 73-74:** "…but also colonize animal and plant tissues (9), emerging particulate pollutants such as microplastics (10)".
>
> **Lines 120-121:** "…also in environmental settings such as in soils (39), water plumbing (40), and wastewater treatment systems (41)…".
>
> **Lines 483-484:** "…sparse biofilms such as on soil particle aggregates (26) or surfaces in wastewater treatment processes (41)."

This work seems related to existing microbial ecology research into interaction neighborhoods, which might be a nice connection to make – interaction neighborhoods across scales, considering within-colony plasmid transfer and between-colony transfer.

We agree that there is a close connection with recent work on short-range microbial interactions. We have now added discussion and relevant literature around this connection in in the following lines of the revised manuscript.

> **Lines 475-479**: "Our findings therefore expand the scale at which bacterial interaction neighborhoods are assumed to operate, connecting the processes driven by short-range interactions (66, 67) with those occurring between entire colonies when they expand and collide. This multi-scale perspective aligns with recent calls to connect microscale interaction processes to macroscale spatial organization in microbial ecosystems (68)."

Minor comments

32: define sparse here, e.g. by making "composed of discrete bacterial colonies" a parenthetical.

Changed as recommended.

93: "in addition to nutrient availability" due to host growth, presumably?

We agree, but also due to resource pulses in environmental biofilms such as on soil or sediment surfaces. We now include this and a reference in the following lines of the revised manuscript:

> **Line 95**: "In addition to nutrient availability due to host growth or nutrient pulses (18), …".

129: it's not yet clear why you use the pair of fluorescently labelled conspecifics – consider omitting this information here.

Omitted as recommended.

141: "using a full-factorial design" – it's not clear yet what all the factors are. Either state the factors, or omit this information here.

Omitted as recommended.

155-7: why did you first quantify plasmid dynamics within the donor colony? This is worth justifying better here, especially in the context provided, which is antibiotic treatment/resistance. Under antibiotic treatment, we would expect the plasmid to be at or near 100% prevalence within the colony.

We agree that plasmid loss from the system would be reduced under antibiotic treatment, but in this experiment without antibiotics we expected loss to have a strong effect and to determine plasmid load at the colony edge before collision. We now make this clearer connection in the following lines of the revised manuscript.

> **Lines 159-162**: "We first quantified pAR145 dynamics within the plasmid donor colony prior to its collision with the potential recipient colony, as we expected the processes of intra-colony pAR145 transfer and loss during the growth of the plasmid donor colony to affect the total load and thus transfer of pAR145 into the potential recipient colony."

163: intra?

Changed. Thanks for the careful observation.

185: "based on a prior study" -> "based on Lagido et al.'s model"

Changed as recommended.

188: we would therefore expect this time to vary across host-plasmid pairs, as it would depend on specific plasmid cost and mechanisms of plasmid maintenance. Plasmid maintenance is touched on in the discussion, but might be worth briefly mentioning these two factors here (and worth mentioning host-specific plasmid costs in the discussion).

Changed as recommended in the following lines of the revised manuscript.

> **Lines 193-194:** "This is mainly caused by plasmid maintenance costs and the selective pressures acting on plasmid-encoded traits (47, 48)."
>
> **Lines 534-538:** "plasmid loads vary depending on the metabolic costs that plasmids impose on host cells (47) and on the selective pressures acting on plasmid-encoded traits (48), making it necessary in future studies to include plasmid costs and selective pressures as factors of antibiotic resistance spread in spatially-explicit systems."

208: might be worth listing the factors here (distance x transfer probability x loss probability)

Added as recommended.

224: what is a primary plasmid transfer event?

This is a transfer event from populations in the donor to the recipient colony, in contrast to a secondary transfer event which occurs when cells in the recipient colony that received the

plasmid after primary transfer pass it on to additional cells in their vicinity. We now clarify this in the following lines of the revised manuscript

> **Lines 229-232:** "…the total number of plasmid transfer events between cells within the plasmid donor and potential recipient colonies (referred to hereafter as "primary transfer events") as descriptors of plasmid spread."

Supplementary tables 2-4: I can't tell from the table legends in the supplement what makes the information in these tables different. It would be nice to distinguish them more clearly in the legends so that cross-referencing from the text is easier.

The difference in these tables is that Supplementary Tables 2 and 3 both contain the linear model summaries for the total number of transconjugant and primary transfer events, but the first at the final simulation time step and the latter at a common time step. Supplementary Table 4 displays the summary for the model explaining exclusively secondary transfer events. We now include a short and general description in each table's caption for clarity.

Supplementary figures 4-7: label panel headers (e.g. Di = 80 units), or specify in legends

Added as recommended.

253: "a significant negative effect of the plasmid loss probability through its interaction with distance" – why would there be an interaction with distance for secondary transfer?

This happens as collisions between colonies initially positioned at smaller distances transfer plasmids to the recipient colony earlier, which in turn allow for secondary transfer but conditional to how fast plasmids are purged (lost) from the system. We now clarify this in the following lines of the revised manuscript.

> **Lines 421-424:** "The extent of additional plasmid transfer within the recipient colony thus depends on the interaction between the initial distance and plasmid loss probability, as closer distances allow for more transfer as long as plasmids have not been rapidly purges from the system."

272-3: why would this be the case?

Because the estimates are not substantially different and both are affected in the same negative way, it is difficult to say. One can speculate that distance had a slightly stronger negative effect on total transconjugants than on primary transfer events because primary transfer decreases as the collision boundary becomes increasingly dominated by plasmid-carrying cells, such that contacts between plasmid-carrying and -free cells become increasingly rare, and instead secondary transfer within the recipient keeps spreading the plasmid. This means for example in collisions at very close initial distances primary transfer would eventually decrease due to this exhaustion of plasmid-carrying to plasmid-free cell contacts, whereas total transconjugants would keep increasing due to secondary transfer. Due to the speculative nature of our argumentation, however, we choose not to include it in this manuscript.

288-289: any idea why there would be a threshold colony size where primary transfer slows down?

As mentioned above, primary transfer slows down over time since collision as potential plasmid-carrying to plasmid-free cell contacts become exhausted, because the collision boundary becomes dominated by plasmid-carrying cells only. As above, due to the speculative nature of our argumentation, we choose to not include it in this manuscript.

308-310: is there an ecological interpretation for this?

That plasmid loss had a stronger effect on primary transfer than on total transconjugants is likely because plasmid loss controls how many plasmid-carrying cells are left at the collision boundary, controlling the possibility that primary transfer occurs. High conjugation rates have little effect if plasmids are being lost. Additionally, due to the above-mentioned exhaustion of potential primary transfer events, high transfer rates become less important as this transfer cannot be realized. This is again a complex and speculative discussion point that we would rather not include in the manuscript for clarity.

324-5: why did you hypothesize this? Again, it’s worth providing more motivation here.

Our hypothesis is based on the fact that more intermixed populations correspond to a higher number of contacts between cells of distinct type, such as plasmid-carrying and -free cells. This increases the probability of plasmid spread by reducing the number of redundant contacts between plasmid-carrying cells. This phenomenon has been reported in multiple works which we now include in our explicit motivation for this hypothesis in the following lines of the revised manuscript.

> **Lines 338-341:** “More intermixed plasmid-carrying and -free populations can enable more efficient plasmid transfer than conditions where populations are spatially segregated (19, 20), as the number of contacts between plasmid-carrying and -free cells is higher when they are intermixed.”

330-1: just to check that I’ve understood correctly – in this study, “intra-colony” and “secondary” mean the same thing regarding plasmid transfer, and likewise “inter-colony” and “primary”? Similarly, the process described in lines 375-7 could also be called secondary transfer?

Correct, thanks for spotting this inconsistency. We now replace “intraspecific transfer” with “secondary plasmid transfer” throughout the entirety of the manuscript.

346: “by modulating the efficacy of interspecific plasmid transfer” -> by increasing the number of plasmid-carrying donor cells

Increasing the number of plasmid-carrying donor cells is the main factor but more intermixed conditions also lead to fewer situations where transfer is redundant (transfer into recipients that already received the plasmid). This is what we meant by efficacy of plasmid transfer. We now clarify this in the following lines of the revised manuscript.

> **Lines 361-364**: “Higher spatial intermixing will increase the number of plasmid-carrying donor cells as well as reducing the total number of redundant plasmid transfer events (transfer occurring between donor cells and recipient cells that already have the plasmid).”

375-7: This would be secondary transfer?

Correct. Changed accordingly.

391: how can you say that plasmid transfer, plasmid loss, and colony distance (the three factors you tested) are the primary drivers of plasmid spread when you didn't test other factors? Do you mean that within the scope of the study they are all relevant to plasmid spread (in which case the current statement is misleading)?

Thanks for pointing this out. We now clarify this statement is within the scope of the factors tested in this study in the following lines of the revised manuscript.

> **Lines 403-405**: "By expanding the parameter space, our individual-based modelling simulations demonstrate that, within the scope of this study…"

406-9: it's worth mentioning segregation control, eg. toxin-antitoxin systems, here when talking about mechanisms that maintain plasmids in the absence of environmental selection for their genetic cargo.

Thank you for pointing this out. We now include these mechanisms and reference works describing pre- and post-segregational control in the following lines of the revised manuscript.

> **Lines 424-429**: "The segregation control system of a particular plasmid (50) and its post-segregational regulation through, for example, toxin-antitoxin systems (51) will have a large impact on its temporal persistence (41, 42), while the host-specific cost of the plasmid and mechanisms of plasmid maintenance will also determine how rapidly plasmid-free cells dominate the biomass periphery due to their higher intrinsic growth rate."

424-30: can you explicitly bridge this gap? Presumably the transconjugants not accounted for due to primary transfer are due to secondary transfer, indicating that the plasmid is continuing to spread spatially within the recipient colony.

Correct. Secondary transfer explains why the total number of transconjugants kept increasing while the number of primary transfer events had a decelerating trend. We now state this explicitly in the following lines of the revised manuscript.

> **Lines 452-456**: "After all the potential recipient cells along the collision boundary receive the plasmid, there are no more plasmid-free cells for additional inter-colony plasmid transfer to occur. In our system, the plasmid kept spreading into the recipient colony due to secondary transfer, which is higher in conditions of high spatial intermixing between plasmid-carrying and -free cells."

446-7: "critically depends…" I assume this is in reference to the Figure 6 results, and the "foldedness" of the border observed in the round colonies in panels B and C. Do you know if the borders of high intermixing simulations are longer than the low intermixing borders due to this increased two-dimensional surface area?

Thanks for pointing this out. The "foldedness" of the borders of simulated colonies in Figure 6 is not related to intermixing as cell rows grew in both directions at the rates defined for the

other simulations, which are quite similar between cell types. After modifying the description of the methods and results shown in Figure 6, this should now be clear. The critical influence of intermixing between cell types at the collision boundary is not contingent on this surface area but on the total number of transfer events at the collision boundary, which occur at the start of the simulation.

Also, in the text here do you refer to intermixing of the two types of donor cells, or intermixing between donor and recipient cells?

We refer to intermixing between donor and recipient cells, which is what increases the number of non-redundant transfer events. We now clarify this in the following lines of the revised manuscript.

> **Lines 474-475**: "but critically depends on the spatial intermixing of donor and recipient cells along the collision boundary"

459-60: why would you expect spatial factors to be a more important driver in natural microbiomes than plasmid transfer and loss rates?

We did not mean to imply that spatial factors are more important than intrinsic plasmid properties, but that spatial factors are important in sparse biofilms due to their spatial distribution. We now clarify this in the following lines of the revised manuscript.

> **Lines 483-484**: "In addition to the processes of plasmid transfer and loss…".

468: "even when the plasmid is unstable in all community members" Where is this shown? Is this referring to plasmid loss rates tested (which figure)? More clarification is needed here. This also differs from the experimental source-sink results, where the plasmid is stable in the source host.

We apologize for the confusion here. We refer here to the fact that, as shown in Fig 1A and 1B and in Supplementary Table 1 and Supplementary Figure 1, pAR145 is lost rapidly from the *S. stutzeri* donor colony, but we cannot distinguish segregational loss from the preferential growth of plasmid-free cells. We agree the term "unstable" is not the appropriate descriptor of this phenomenon and this sentence has been removed from the revised manuscript.

468-71: what makes these results different and interesting compared to plasmid research that focuses on conjugation/transfer rates isn't coming through enough here. If you can make this clear, it will increase the impact of the work.

We greatly appreciate this suggestion and it prompted us to search the literature further. Interestingly, we identified a study that applied a metacommunity model to predict the spread of plasmids among biomass patches (Xue, Wenzhi, et al. "Spatial entropy drives the maintenance and dissemination of transferable plasmids." Molecular Systems Biology 21.7 (2025): 856-869.) This study allows us to better emphasize the distinct knowledge field that our findings contribute to, and to better connect it to the discussion on source-sink dynamics. We now discuss this in the following lines of the revised manuscript.

> **Lines 490-502**: "In fact, a recent study modelled plasmid dynamics between biomass patches in simulated metacommunities, finding that costly plasmids are better

maintained in the system when patches are unevenly distributed (72). This allows patches to grow in density and act as plasmid reservoirs for additional transfer upon contact with other patches. This is akin to source-sink dynamics, which are a way by which a stable plasmid donor ensures the plasmid is maintained in unstable plasmid recipients by frequent transfer (14). Our study expands such findings by showing that the physical proximity between sources and sinks determines plasmid spread between biomass patches, and that consideration of the spatial intermixing of plasmid-carrying cells within patches is a yet unrecognized factor driving plasmid spread. By combining our predictive framework with a metacommunity model that explicitly considers the spatial distribution of biomass patches, it becomes increasingly feasible to establish predictions of plasmid fate in sparse biofilms."

491-4: did you test this here? Is this referring to existing work (citation(s))? You haven't made the link to plasmid cost clear enough here, nor the link to the work presented here.

We agree that this was unclear. We have now reformulated the sentence to distinguish plasmid cost from selective pressures on plasmid-encoded traits, as these are independent aspects even though they can lead to similar outcomes (i.e. plasmid maintenance or loss from the system). We now discuss this and included relevant literature citations to support this distinction in the following lines of the revised manuscript.

Lines 534-538: "Finally, plasmid loads vary depending on the metabolic costs plasmids impose on host cells (47) and on the selective pressures acting on plasmid-encoded traits (48), making it necessary in future studies to include plasmid costs and selective pressures as factors of antibiotic resistance spread in spatially explicit systems."

524: please include the pure culture growth conditions (media and temperature).

Added as recommended.

652: suggestion to include citations for software and packages

Added as recommended for R and the MASS package.

Figure 1:
Please explain more clearly how to interpret the fluorescence that we see. In the images, I see red, green, purple, and blue.

We now clarify how the different colors originate in the following lines of the revised manuscript

Lines 990-994: "one strain expresses red fluorescent protein from the chromosome and cyan fluorescent protein from pAR145 and appears magenta. The other strain only expresses green fluorescent protein from the chromosome. If pAR145 transfers into the strain expressing green fluorescent protein, then it appears cyan."

In panel C, how did you distinguish the types of HGT indicated by the different arrows? Please explain in the methods as well.

During strain construction and testing of individual growth rates we could see that plasmid-carrying *E. coli* performs HGT with its plasmid-free counterparts, and it is visible that transfer is occurring from *S. stutzeri* to *E. coli*. The way to distinguish this is through changes in color as pAR145 contains cyan fluorescent protein, which we now clearly describe in the figure caption and methods in the following lines of the revised manuscript.

> **Lines 604-606**: "These experiments allowed us to quantify the transfer of pAR145 from plasmid-carrying cells in the *S. stutzeri* donor colony (visualized as cyan or magenta) into plasmid-free cells in the *E. coli* recipient colony (visualized as blue cells)."
>
> **Lines 997-1001**: "a blue patch located along the collision boundary, which corresponds to *E. coli* cells that acquired pAR145 via inter-colony transfer from pAR145-carrying *S. stutzeri* cells (white arrow). Yellow arrows indicate the spread of pAR145 into the recipient colony, which can happen via transfer among *E. coli* cells as well as additional growth."

Figure 2:
Can something be done to increase the visibility of the blue patches in panel A?

We now include the revised panel A with additional insets showing a magnified blue patch area.

For the overlapping dots in panel B, try reducing the alpha so that layered points show up darker.

Modified as recommended.

Out of curiosity and ecological interest, can you explain what is happening at the collision edge to give it the arc shape? Is the Pseudomonas colony swarming/replicating around the edge of the E. coli colony, or is the E. coli colony replicating into the Pseudomonas colony?

The arc shape of the boundary between colonies that collide happens when one of them, in this case the *E. coli* one, has a lower relative growth rate to the other colony. Both are replicating, but we think that nutrient limitation rapidly stops this growth at the collision edge, with physical uplifting and other processes creating the patterns we see, with the additional HGT among *E. coli* cells causing the protrusion of the blue area into the colony.

Figure 4:
In Panel A it says D, not Di.

Corrected as identified.

At lines 949 & 952, "(blue in A)".

Corrected as identified.

At lines 949 & 951, "initial distance (Di)".

Corrected as identified.

Line 954: the colors are from B & C

Corrected as identified.

Figures S13 and S14: suggestion to refer to them in the results and give them a little interpretation rather than only in the discussion. Also, the A and B are difficult to see against the dark purple.

We now include a short description of these figures in the results section and modified the visualization of the letters as suggested.

> **Lines 263-269**: "We also found that conditions of low plasmid transfer and high plasmid loss probabilities were conducive for the appearance of rare plasmid spread events in collisions occurring at large initial distances (Supplementary Fig. 10A; Supplementary Fig. 11A). By contrast, conditions of high plasmid transfer and low plasmid loss probabilities in collisions occurring at short initial distances lead to highly predictable plasmid spread but with high absolute variance among replicates (Supplementary Fig. 10A; Supplementary Fig. 11A)."

Reviewer #2 (Comments for the Author):

The manuscript "Spatial constraints determine the spread of plasmid-encoded antibiotic resistance between bacterial colonies" explores the influence of plasmid transfer and loss in the spread of antibiotic resistance on surfaces, particularly concerning the distance between donor and recipient populations. By combining structured colony experiments with an individual-based modelling approach, the study shows how important spatial organization is for plasmid conjugation and antimicrobial resistance dissemination.

Overall, this study offers a useful quantitative analysis that connects plasmid transfer, plasmid loss, and the spatial structure of bacterial colonies. The integration of experiments and individual-based modelling is coherent and well executed, and it refines current understanding of how spatial organization shapes the spread of antibiotic resistance. The study also addresses its limitations, such as cell type, compatibility for conjugation, and simplifications in the model.

The impact and general applicability of this study could increase by broadening the modelling framework beyond its current parameters. Specifically, finding critical thresholds for crucial factors like plasmid cost, transfer probability, temporality, and spatial separation would allow for a broader assessment of conjugation limits. Such analysis (already sketched in Fig. 5) would make this work more relevant as a widely applicable predictive tool.

We appreciate the constructive comments and assessment of the work. We do believe expanding the range of parameters could identify thresholds that improve the predictive usefulness of the approach, in particular the addition of plasmid cost. This would also allow a more direct connection with the fitness effects on plasmid-carrying cells under antibiotic exposure, which we see as a main potential application. However, our objective here was to first combine experiments and modelling to identify the key factors behind inter-colony plasmid

spread, which already required a significant computational effort. We think the careful quantitative evaluation of fitness costs would require a more focused simulation study, with fewer parameters initially (e.g. just tuning transfer, loss, and cost first, as the effect of initial distance could be investigated as a function of plasmid load at the collision boundary after). Thus, while we completely agree with the reviewer, we believe these efforts would justify a new study rather than merely being added to this study.

Furthermore, it would be beneficial to discuss in depth the model predictions shown in Fig. 6.

We agree that we did not sufficiently describe these results, which was also pointed out by Reviewer 1. We have now expanded the discussion on the influence of spatial intermixing on the spread of plasmids in the following lines of the revised manuscript.

> **Lines 440-445**: "…affects both the total plasmid load and the degree of intermixing of the plasmid-free and -carrying cells in a potential recipient colony. There is ample evidence that more intermixed populations lead to a larger number of cell-cell contacts between distinct cell types (in this case plasmid-free and -carrying types) (e.g. (19, 58). This has, for example, consequences for the structuring of communities via metabolic exchange (59) or contact-dependent killing (60)."
>
> **Lines 463-465**: "Surface evaporation can also increase the intermixing between plasmid-carrying and -free cells and promote plasmid spread (64, 65)."
>
> **Lines 496-502**: "Our study expands such findings by showing that the physical proximity between sources and sinks determines plasmid spread between biomass patches, and that consideration of the spatial intermixing of plasmid-carrying cells within patches is a yet unrecognized factor driving plasmid spread. By combining our predictive framework with a metacommunity model that explicitly considers the spatial distribution of biomass patches, it becomes increasingly feasible to establish predictions of plasmid fate in sparse biofilms."

The spatial organization of the donor colony shown in Fig. 1 suggests that the system may not be accurately described by a single static mixing pattern throughout colony development. The donor colony's periphery shows a low-intermixing structure, with plasmid-bearing cells being located in relatively distant sectors, while the inner region of the colony shows a higher degree of intermixing and more plasmid carriers. This observation indicates that conjugation dynamics could vary over time during colony collision, reflecting changes in spatial structure as the colony grows and matures.

Thank you for this observation. True, in this system the region of the plasmid donor colony that has not lost the plasmid at the point of collision is also the one displaying the highest intermixing. We were not able to separate these two factors experimentally, but that is why we included the simulations in Fig. 6, where we were able to manipulate intermixing with (panel B) and without (panel C) and quantify how it affects plasmid load at the collision boundary. From this one can connect to the temporal aspect: in systems where plasmids are not rapidly lost from the donor colony, additional transfer after initial transfer from the well-mixed colony region will be a function of the degree of intermixing achieved at the periphery, so indeed conjugation dynamics will vary non-linearly over time. We now elaborate on this in the following lines of the revised manuscript and within a new discussion paragraph as reported in the last response.

> **Lines 503-508**: "As shown by our experiments and simulations, the spatial distribution of plasmid-carrying and -free cells changes over time due to the de-mixing of populations during surface growth. This is the result of stochastic fluctuations in the boundaries between growing populations that results in strong genetic drift (22, 73). This implies that the degree of intermixing between populations is a time-dependent process, such that collisions occurring before de-mixing are predicted to increase plasmid spread."

It would be interesting to investigate whether including the temporal evolution of spatial organization in the modelling during colony collision could capture potential shifts in conjugation dynamics.

In relation to the previous comment, this is something that could be achieved by changing the simulation parameters to lower the static plasmid fitness cost we experimentally measure. This is because under the current setup, the area where plasmids are still present coincides with the highest intermixing, so this would require a significant change in the modelling parameters. We therefore suggest that such a dynamical analysis would best be reserved for a new manuscript dedicated on that topic.

An extensive discussion on how low- and high-intermixing regimes develop and how timing affects conjugation dynamics would enhance the understanding of the observed spatial dynamics.

We fully agree, and we now include additional discussion of the topic in the manuscript. We specifically discuss the fundamental processes behind population de-mixing and work that has previously assessed the interplay between intermixing and plasmid spread over time. We elaborate this discussion in the following lines of the revised manuscript:

> **Lines 503-515**: "As shown by our experiments and simulations, the spatial distribution of plasmid-carrying and -free cells changes over time due to the de-mixing of populations during surface growth. This is the result of stochastic fluctuations in the boundaries between growing populations that results in strong genetic drift (22, 73). This implies that the degree of intermixing between populations is a time-dependent process, such that collisions occurring before de-mixing are predicted to maximize plasmid spread. Previous work has shown that the timing of antibiotic administration in surface-associated bacterial systems can determine plasmid spread by creating a positive selective pressure when plasmids are still readily transferring within the community due to high intermixing (74). As intermixing decreases over time, the spread of plasmid-mediated antibiotic resistance becomes increasingly confined to fewer regions where plasmid-carrying and -free cells are still in contact. Considering the temporal dynamics of population de-mixing is therefore important for predicting plasmid spread into neighboring biomass."

Re: mSystems00531-26R1 (**Spatial constraints determine the spread of plasmid-encoded antibiotic resistance between bacterial colonies**)

Dear Dr. Josep Ramoneda:

Your manuscript has been accepted, and I am forwarding it to the ASM production staff for publication. Your paper will first be checked to make sure all elements meet the technical requirements. ASM staff will contact you if anything needs to be revised before copyediting and production can begin. Otherwise, you will be notified when your proofs are ready to be viewed.

Data Availability: ASM policy requires that data be available to the public upon online posting of the article, so please verify all links to sequence records, if present, and make sure that each number retrieves the full record of the data. If a new accession number is not linked or a link is broken, provide production staff with the correct URL for the record. If the accession numbers for new data are not publicly accessible before the expected online posting of the article, publication may be delayed; please contact ASM production staff immediately with the expected release date.

Publication Fees: For information on publication fees and which article types have charges, please visit our website. We have partnered with Copyright Clearance Center (CCC) to collect author charges. If fees apply to your paper, you will receive a message from no-reply@copyright.com with further instructions. For questions related to paying charges through RightsLink, please contact CCC at ASM_Support@copyright.com or toll free at +1-877-622-5543. CCC makes every attempt to respond to all emails within 24 hours.

ASM Membership: Corresponding authors may join or renew ASM membership to obtain discounts on publication fees. Need to upgrade your membership level? Please contact Customer Service at Service@asmusa.org.

Embargo Policy: A press release may be issued as soon as the manuscript is posted on the mSystems Latest Articles webpage. The corresponding author will receive an email with the subject line "ASM Journals Author Services Notification" when the article is available online.

Cover Image Submissions: If you would like to submit a potential Cover Image, please email a file and a short legend to msystems@asmusa.org. Please note that we can only consider images that (i) the authors created or own and (ii) have not been previously published. By submitting, you agree that the image can be used under the same terms as the published article. Image File requirements: TIF/EPS, 7.5 inches wide by 8.25 inches tall (at least 2,250 pixels wide by 2,475 pixels tall), minimum 300 dpi resolution (600 dpi preferred), RGB, and no figure elements, e.g., arrows or panel labels. The legend should be a short description of the image, 1-2 sentences recommended. Please use the ASM Journals Cover Image Sizing Tool (https://journals.asm.org/pb-assets/html/asm-cover-sizing-tool.html) to ensure that your proposed cover image meets our size requirements.

Thank you for submitting your paper to mSystems.

Sincerely,
William Harcombe
Editor
mSystems

Reviewer #1 (Comments for the Author):

The authors have fully addressed my comments and the revised manuscript is clear and compelling.

Reviewer #2 (Comments for the Author):

The authors have addressed most of the suggested changes and have clarified several sections of the manuscript, making the study easier to follow and better highlighting its relevance. I believe that some of the remaining questions can be answered in future studies.
