# Supplementary material for: Spatial constraints determine the spread of plasmid-encoded antibiotic resistance between bacterial colonies
Source: mSystems. 2026 Aug 28;11(9):e00531-26. doi: 10.1128/msystems.00531-26 (PMC13595988; doi:10.1128/msystems.00531-26)
Supplement: Supplementary file 2 [file msystems.00531-26-s0001.docx]

**Supplementary materials**

**Supplementary Table 1.** Quantification of relative growth rates of *S. stutzeri* A1601-*ech* with pAR145 and *S. stutzeri* A1601-*gfp*. $s$ is the selective advantage, or in our case the cost, that the plasmid bestows in the absence of chloramphenicol; $R_{b}$ is the radius of the circle generated by the arc at the boundary; $l$ is the distance between centroids of two colonies. $R_{b}$ and $l$ are expressed in µm.

| **Replicate** | $\boldsymbol{s}$ | $\boldsymbol{R}_{\boldsymbol{b}}$ | $\boldsymbol{l}$ |
| --- | --- | --- | --- |
| 1 | 0.834 | 20.755 | 26.823 |
| 2 | 0.84 | 23.075 | 29.868 |
| 3 | 0.827 | 23.076 | 29.524 |
| 4 | 0.83 | 23.074 | 29.580 |

**Supplementary Table 2.** Full model summary at the final simulation time step. Summary of the generalized linear model for testing the full effects of the plasmid transfer probability, plasmid loss probability, and initial distance between colonies on the total number of transconjugants and the number of primary plasmid transfer events after collision of a plasmid donor and a potential recipient colony. We performed a total of 1500 simulations. Full model formula: *Y ~ Transfer probability + Loss probability + Distance + Transfer:Loss + Transfer:Distance + Loss:Distance + Transfer:Loss:Distance* (n = 10). Coefficients (*b*) are in log-scale due to the negative binomial log-link.

|  | **Total number of transconjugant cells** | | | **Total number of primary transfer events** | | |
| --- | --- | --- | --- | --- | --- | --- |
| **Factor** | ***b*** | **SE** | ***P*** | ***b*** | **SE** | ***P*** |
| Transfer probability | 614.053 | 183.635 | 0.0008 | −157.970 | 117.71 | 0.18 |
| Loss probability | 0.266 | 0.942 | 0.777 | 1.913 | 0.72 | 0.008 |
| Distance | −0.038 | 0.005 | <0.001 | −0.028 | 0.003 | <0.001 |
| Transfer x Loss probability | 365.247 | 324.232 | 0.26 | −66.860 | 231.7 | 0.773 |
| Distance x Transfer probability | 1.455 | 1.743 | 0.404 | 4.839 | 1.134 | <0.001 |
| Distance x Loss probability | −0.036 | 0.009 | <0.001 | −0.050 | 0.007 | <0.001 |
| Distance x Transfer x Loss probability | 2.255 | 3.137 | 0.472 | 6.489 | 2.308 | 0.005 |

**Supplementary Table 3.** Full model summary at a common simulation time step. Summary of the generalized linear model for testing the full effects of the plasmid transfer probability, plasmid loss probability, and initial distance between inocula on the total number of transconjugants and of primary plasmid transfer events after collision of a plasmid donor and a potential recipient colony at a fixed simulation time point of 640. We performed a total of 1500 simulations and quantified the total number of transconjugant cells and the number of accumulated plasmid transfer events at a fixed simulation time point of 640. Full model formula: *Y ~ Transfer probability + Loss probability + Distance + Transfer:Loss + Transfer:Distance + Loss:Distance + Transfer:Loss:Distance* (n = 10). Coefficients (*b*) are in log-scale due to the negative binomial log-link.

|  | **Total number of transconjugants** | | | **Total number of primary transfer events** | | |
| --- | --- | --- | --- | --- | --- | --- |
| **Factor** | ***b*** | **SE** | ***P*** | ***b*** | **SE** | ***P*** |
| Transfer probability | 594.228 | 184.965 | 0.001 | −93.073 | 117.724 | 0.429 |
| Loss probability | 0.398 | 0.935 | 0.67 | 1.551 | 0.707 | 0.028 |
| Distance | −0.030 | 0.005 | <0.001 | −0.022 | 0.003 | <0.001 |
| Transfer x Loss probability | 218.048 | 323.661 | 0.501 | −66.175 | 228.726 | 0.772 |
| Distance x Transfer probability | 1.335 | 1.752 | 0.446 | 4.093 | 1.129 | <0.001 |
| Distance x Loss probability | −0.037 | 0.009 | <0.001 | −0.045 | 0.007 | <0.001 |
| Distance x Transfer x Loss probability | 3.902 | 3.114 | 0.21 | 6.365 | 2.256 | 0.005 |

**Supplementary Table 4.** Full model summary for secondary plasmid transfer events. Summary of the generalized linear model for testing the full effects of the plasmid transfer probability, plasmid loss probability, and initial distance between inocula on the total number of secondary plasmid transfer events after collision of a plasmid donor and a potential recipient colony. We performed a total of 1500 simulations. Full model formula: *Y ~ Transfer probability + Loss probability + Distance + Transfer:Loss + Transfer:Distance + Loss:Distance + Transfer:Loss:Distance* (n = 10). Coefficients (*b*) are in log-scale due to the negative binomial log-link.

|  | **Total number of secondary transfer events** | | |
| --- | --- | --- | --- |
| **Factor** | ***b*** | **SE** | ***P*** |
| Transfer probability | 656.721 | 208.295 | 0.002 |
| Loss probability | −0.247 | 1.1 | 0.823 |
| Distance | −0.047 | 0.006 | <0.001 |
| Transfer x Loss probability | 540.515 | 372.661 | 0.147 |
| Distance x Transfer probability | 2.805 | 1.988 | 0.158 |
| Distance x Loss probability | −0.030 | 0.011 | 0.006 |
| Distance x Transfer x Loss probability | 0.071 | 3.634 | 0.984 |

**Supplementary Table 5.** Specifications of the strains and plasmid used in this study.

| Strain | Relevant characteristics | Reference |
| --- | --- | --- |
| *S. stutzeri* A1601-*egfp* | *S. stutzeri* A1501 (wild type) with Δ*comA* and mini-Tn7T-LAC-Gm-*egfp*; Gm^R^, *egfp*^+^ | 82,83 |
| *S. stutzeri* A1601-*ech* | *S. stutzeri* A1501 (wild type) with Δ*comA* and mini-Tn7T-LAC-Gm-*ech*; Gm^R^, *ech*^+^ | 82,83 |
| *E. coli* DH5α | F2 *supE44 lacU169 (w80lacZDM15) hsdR17 recA1 endA1 gyrA96thi-1 r elA1* | 85 |
| pAR145ecfp | pSU2007 *aph::cat*-PA1/04/03-*cfp*⁄-T0 | 84 |

**Supplementary Figure 1.** Loss of pAR145 in the absence of chloramphenicol during colony growth. (A) We started the colony experiment with a single pAR145-carrying strain of *S. stutzeri* A1601 that encodes for *echerry* on its chromosome. It therefore initially displays both red fluorescence from the chromosome and blue fluorescence from pAR145 and appears the composite color magenta. The white arrow identifies a pAR145 loss event indicated by a change in color from magenta to red. (B) We performed the collision experiment between *S. stutzeri* A1601-*ech* carrying pAR145 (magenta) and *S. stutzeri* A1601-*ech* (red). We inoculated the two strains at the same time at a distance of approximately 3 mm away from each other. We took images after 96 hours of incubation at room temperature. The smaller size of the pAR145-carrying colony indicates a slower growth rate, demonstrating that carrying pAR145 is costly in the absence of chloramphenicol.

**Supplementary Figure 2**. Collision assay of *S. stutzeri* A1601-*ech* carrying pAR145 and *S. stutzeri* A1601-*gfp*. We drew the larger circle based on the arc generated between the two colonies. *X_0_* and *O* are the centroids of the derived circle and the green colony, respectively. $l$ is the distance between *X_0_* and *O*. $R_{b}$ is the radius of the derived circle. We use following equation to calculate the relative growth rate $s$.

$$R_{b}=l\frac{1+s}{s(2+s)}$$

**Supplementary Figure 3.** Collision assays of *S. stutzeri* A1601-*gfp*, *S. stutzeri* A1601-*ech*, and *E. coli* DH5α either carrying or not carrying pAR145. The straight yellow lines at the collision interfaces indicate equal growth rates of the two strains. (A) Collision assay of *S. stutzeri* A1601-*ech* carrying pAR145 (plasmid donor) and *S. stutzeri* A1601-*gfp* carrying pAR145. (B) Collision assay of *S. stutzeri* A1601-*ech* (plasmid donor after losing pAR145) and *S. stutzeri* A1601-*gfp*. (C) Collision assay of *E. coli* DH5α (black) and *E. coli* DH5α carrying pAR145 (blue).

**Supplementary Figure 4.** Interactive effects of plasmid transfer and loss probabilities and initial distance between inocula on the total number of transconjugants. Numbers on each panel indicate the initial distances between the inocula at the start of the simulation (d = 80-90-100-110-120-130).

**Supplementary Figure 5.** Interactive effects of plasmid transfer and loss probabilities and initial distance between inocula on the total number of primary transfer events. Numbers on each panel indicate the initial distances between the inocula at the start of the simulation (d = 80-90-100-110-120-130).

**Supplementary Figure 6.** Interactive effects of plasmid transfer and loss probabilities and initial distance between inocula on the total number of primary transfer events. Numbers on each panel indicate the initial distances between the inocula at the start of the simulation (d = 80-90-100-110-120-130).

**Supplementary Figure 7.** Interactive effects of plasmid transfer and loss probabilities and initial distance between inocula on the total number of primary transfer events. Numbers on each panel indicate the initial distances between the inocula at the start of the simulation (d = 80-90-100-110-120-130).

**Supplementary Figure 8.** Effect of plasmid transfer and loss probabilities on plasmid spread between colliding colonies at a fixed simulation time step of 640. (A) Total number of transconjugant cells as a function of the plasmid transfer probability. (B) Total number of primary plasmid transfer events between the plasmid donor and potential recipient colonies as a function of the plasmid transfer probability. (C) Total number of transconjugant cells as a function of the plasmid loss probability. (D) Total number of primary plasmid transfer events between the plasmid donor and potential recipient colonies as a function of the plasmid loss probability (n = 10).

**Supplementary Figure 9.** Effect of plasmid transfer and loss probabilities on the total number of secondary plasmid transfer events within the potential recipient colony. (A) Total number of secondary plasmid transfer events at the end of the simulation as a function of the plasmid transfer probability. (B) Total number of transconjugant cells as a function of the total population size, colored by plasmid transfer probability as defined in (A). (C) Total number of secondary plasmid transfer events at the end of the simulation as a function of the plasmid loss probability. (D) Total number of secondary plasmid transfer events as a function of the total population size, colored by the plasmid loss probability as defined in (C) (n = 10).

**A**

**B**

**A**

**B**

**B**

**B**

**A**

**A**

**Supplementary Figure 10.** Variation in the total number of transconjugants and of primary transfer events across replicate simulations of all treatments, shown as (A) Coefficient of variation (CV), and (B) variance (n = 10). Note that high CVs in both the total number of transconjugants and of primary transfer events are observed in simulations where due to high initial distances between microcolonies or due to high plasmid loss probabilities transfer events are rare, such that a few simulations where these occur strongly increase this metric. High variance in the total number of transconjugants and of primary transfer events is instead mostly observed in simulations where high plasmid transfer probabilities or short initial distances lead to large numbers of transconjugants. Letters A and B on the heatmap correspond to the panels in Supplementary Fig. 15 that illustrate the complementary information provided by these metrics.

**Supplementary Figure 11.** Simulation images at the final simulation time point showing the variability of outcomes for selected treatments across all replicates (n = 10). (A) Simulation images corresponding to conditions leading to low variance in total transconjugants and primary transfer events between microcolonies but with high coefficient of variation (CV) due to rare transfer events. (B) Simulation images corresponding to conditions leading to high variance in total transconjugants and primary transfer events between microcolonies but with low CV due to high mean numbers of transconjugants and of transfer events. We avoided depicting cases of extreme CV in (A) as these were driven by single rare events involving too few cells to be clearly visible. Transfer events in (A) are indicated with white arrows.

**Supplementary Figure 12.** Effect of the initial distance between colonies on plasmid spread at a fixed simulation time step of 640. (A) Total number of transconjugant cells as a function of the distance between inocula. (B) Total number of primary plasmid transfer events between the plasmid donor and potential recipient colonies as a function of the distance between inocula (n = 10).

**Supplementary Figure 13.** Effect of the initial distance between colliding colonies on the total number of secondary plasmid transfer events within the potential recipient colony. (A) Total number of secondary plasmid transfer events at the end of the simulation as a function of the distance between inocula. (B) Total number of secondary plasmid transfer events as a function of the total population size (n = 10).

**Supplementary Figure 14.** Relative importance of the plasmid transfer and loss probabilities as a function of the initial distance between inocula on the total number of secondary plasmid transfer events within the potential recipient colony (n = 10).
